# Dissecting tRNA-derived fragment complexities using personalized transcriptomes reveals novel fragment classes and unexpected dependencies

**DOI:** 10.18632/oncotarget.4695

**Published:** 2015-07-06

**Authors:** Aristeidis G. Telonis, Phillipe Loher, Shozo Honda, Yi Jing, Juan Palazzo, Yohei Kirino, Isidore Rigoutsos

**Affiliations:** ^1^ Computational Medicine Center, Sidney Kimmel Medical College at Thomas Jefferson University, Philadelphia, PA, USA; ^2^ Department of Pathology Anatomy and Cell Biology, Sidney Kimmel Medical College at Thomas Jefferson University, Philadelphia, PA, USA

**Keywords:** tRNA fragment, human genome, nuclear tRNA, mitochondrial tRNA, Argonaute

## Abstract

We analyzed transcriptomic data from 452 healthy men and women representing five different human populations and two races, and, 311 breast cancer samples from The Cancer Genome Atlas. Our studies revealed numerous constitutive, distinct fragments with overlapping sequences and quantized lengths that persist across dozens of individuals and arise from the genomic loci of all nuclear and mitochondrial human transfer RNAs (tRNAs). Surprisingly, we discovered that the tRNA fragments' length, starting and ending points, and relative abundance depend on gender, population, race and also on amino acid identity, anticodon, genomic locus, tissue, disease, and disease subtype. Moreover, the length distribution of mitochondrially-encoded tRNAs differs from that of nuclearly-encoded tRNAs, and the specifics of these distributions depend on tissue. Notably, tRNA fragments from the same anticodon do not have correlated abundances. We also report on a novel category of tRNA fragments that significantly contribute to the differences we observe across tissues, genders, populations, and races: these fragments, referred to as i-tRFs, are abundant in human tissues, wholly internal to the respective mature tRNA, and can straddle the anticodon. HITS-CLIP data analysis revealed that tRNA fragments are loaded on Argonaute in a cell-dependent manner, suggesting cell-dependent functional roles through the RNA interference pathway. We validated experimentally two i-tRF molecules: the first was found in 21 of 22 tested breast tumor and adjacent normal samples and was differentially abundant between health and disease whereas the second was found in all eight tested breast cancer cell lines.

## INTRODUCTION

A major characteristic of the last decade is the explosion in the amount of genomic, transcriptomic, epigenomic, and other “–omic” data that can be generated for a given individual, healthy or patient. Not surprisingly, the ensuing data tsunami enabled invaluable interdisciplinary and integrative analyses that linked molecular signatures to phenotypes and diseases [1, 2]. At the same time it recast the definition of disease and the practice of treatment giving both an increasingly more individualized character. Where the microarray technology of the late 90's and early 00's deepened our understanding of disease, deep-sequencing technology has allowed us to refine, and frequently redefine, our views. In fact, the technological improvements in deep-sequencing of the last several years have helped establish new categories of molecular players, particularly non-coding RNAs (ncRNAs), with critical roles in many contexts for which we thought we had reasonably comprehensive descriptions [3, 4]. Such discoveries have important implications for our practice of individualized therapy, especially since they can reveal molecular differences that depend on gender, population and race, all three being patient attributes whose relevance in deciding a therapeutic regime has traditionally not been taken into account.

TRNAs are ancient ncRNAs with a central role in the process of translation of a messenger RNA (mRNA) into an amino acid sequence. As such, tRNAs are present in archaea, bacteria, and eukaryotes. The conventional understanding had been that genomic loci harboring tRNAs produce a single precursor transcript that is processed to produce the mature tRNA. Recent evidence, however, suggests that “tRNA fragments” represent a novel and potentially important group of ncRNAs. However, the knowledge about their biogenesis, their roles and their potential function remains limited [5–8]. Studies with human cell lines have shown that tRNAs can be cleaved at the anticodon loop to produce “tRNA halves” that are 30-35 nt in length a process that seems to be facilitated by the enzyme Angiogenin following induction of stress [9–13]. tRNA fragments (‘tRFs’) have also been found to originate from cleavage of either the mature tRNA or the tRNA precursor molecule. In the latter case, RNase Z cleaves the 3′ part of the tRNA precursor as part of the maturation process with the resulting fragment also being considered a tRF [14, 15]. tRFs that are derived from mature tRNAs emerge after cleavage at either the D-loop (giving rise to 5′-tRFs) or the T-loop (giving rise to 3′-tRFs with the CCA addition present) and are approximately 20 nt long [15–17]. Further investigation into the enzymes responsible for the fragments has shown that the process is Dicer-dependent in several organisms [14, 18–20] but not in the mouse, the fruitfly *Drosophila melanogaster*, or the yeast *Schizosaccharomyces pombe* [21]. The generation of fragments is also Angiogenin-dependent (cleaving the tRNA at the T-loop) [19] and RNase-Z-dependent (producing 5′-tRFs) [14].

The available evidence indicates that tRFs are not random degradation products [15, 18]. Indeed, some 3′-tRFs are loaded on Argonaute thereby regulating RNA abundance [14, 21] and affecting physiological processes like cell growth [12], cell proliferation [15] and cellular responses to DNA damage [20]. tRFs have been shown to have regulatory roles in translation initiation [10] and stress granule formation [22]. 3′-tRFs have also been described to emerge in human MT4 T-cells after HIV infection from the host cell [23]. Further adding to the likelihood that they are not random in nature is the fact that tRFs have been described in mouse [24, 25], yeast [26, 27], the protozoan *Giardia lamblia* [28], *Tetrahymena thermophila* [29, 30], the bacterium *Streptomyces coelicolor* [31], and the archaeon *Haloferax volcanii* [32].

The idiosyncrasies of tRNA genomics necessitate that special computational provisions be made when building an analytical pipeline for the study of tRFs using next-generation sequencing datasets. Additionally, from an experimental perspective, conventional schemes are of little assistance and new biochemical approaches are needed to specifically amplify tRFs of interest. Lastly, from a patient perspective, it is important to investigate the extent and nature of functional tRFs that are contributed to the pool of active molecules in a given tissue and how they change between men and women, between races, and between individuals who belong to the same race but different population groups.

Below, we describe our interdisciplinary investigations of these questions and report on the findings that resulted from our study of two large datasets: lymphoblastoid cell lines derived from 452 men and women representing five human populations [33] and 311 breast samples from the BRCA repository of The Cancer Genome Atlas (TCGA) at the National Institutes of Health (NIH) [34].

## RESULTS

First, we describe the technical and algorithmic hurdles that we needed to overcome in order to properly analyze deep-sequencing data arising from nuclear and mitochondrial tRNA loci. We follow by describing the new molecular category of i–tRFs, and our discovery of the multiple dependencies of the tRNA fragments on disease and on the characteristics of individuals. We conclude by reporting on our experimental validation of two i-tRFs in 22 normal and breast cancer samples and in eight breast model cell lines.

### Background considerations

The proper analysis of tRNA sequences requires several considerations that go well beyond what is typically done when one maps RNA-seq data for the purpose of e.g., profiling the expression of miRNAs or mRNAs. Among other things, these considerations stem from the fact that tRNAs are repeat elements. Considering the hierarchy at hand, where each amino acid (at the top of the pyramid) has multiple associated anticodons (*isoacceptors*) and each anticodon has multiple associated genomic instances (*isodecoders*, at the bottom of the pyramid), our analyses seek to unravel for each tRNA fragment details at the *lowest possible level* of the hierarchy. We stress that due to the nature of the sequences at hand this goal is inherently unattainable in some instances. As many isodecoders have indistinguishable sequences, we report our results at the *anticodon* level, an intermediate level between isoacceptors and isodecoders. We also keep track of the genomic origins of all fragments reported (see the Notation section in Materials and Methods).

*Nota bene*: In what follows, all shown sequences are given in 5′→3′ orientation. Also, the terms “tRNA-derived fragments”, ”tRNA fragments”, “tRFs” and “fragments” will be used interchangeably throughout the text. We use the terms “nuclear” and “mitochondrial” tRNAs to refer to the nuclearly-encoded and mitochondrially-encoded tRNAs, respectively. However, this distinction of the origin of the DNA precursor template may not be entirely accurate from a biological standpoint: as we recently reported [35, 36], mitochondrially-encoded tRNAs have numerous lookalikes in the nuclear genome (see below and also Discussion).

*Nomenclature:* in what follows, we will be recognizing “three tRNA regions” and “three types of tRNA fragments.” The three tRNA regions are “*+1*,” “*internal*,” and “*CCA-ending*” and give rise to “5′-tRFs,” “i-tRFs,” and “3′-tRFs” respectively.

*Data availability:* In order to facilitate research on tRNA fragments, we include all the data matrices used in this study as Supplementary Tables. In addition, we make the data available through our website at https://cm.jefferson.edu/tRNA-fragments-2015/.

### Mapping with mismatches will generate erroneous results

Multiple sequence alignments of the genomic copies for a given anticodon reveal many instances of sequence segments that are shared among these copies and can be made to look like one another if one permits a small number of either insertions/deletions (indels), or replacements. These segments can occur anywhere across the length of the mature tRNA, and thus be present in tRNA fragments. Moreover, these segments can occur in the sequences of *distinct* anticodons of the *same* amino acid. Consequently permitting indels or replacements during read mapping will misidentify the genomic origin of a read and lead to erroneous results. As the following example highlights, problems can occur even if we excluded indels and allowed a single replacement. Let us consider the 5′-tRF GGGGAATTAGCTCAAG-T-GGTAGAGCGCTTGCT which appears at five genomic locations, all of which are AlaAGC tRNA instances. By contrast, the 32 nt sequence GGGGAATTAGCTCAAG-C-GGTAGAGCGCTTGCT, which differs from the previous one at a single location (T→C), is a 5′-tRF of AlaAGC but appears at two *different* genomic locations that are distinct from the previous five. If we were to allow for read mapping with a single mismatch these two distinct 5′-tRF molecules would become indistinguishable confounding any transcriptional differences that potentially exist among the seven loci where GGGGAATTAGCTCAAG-N-GGTAGAGCGCTTGCT is found. The problem is accentuated further when working with the typically shorter reads that are contained in “short” RNA-seq datasets. The 22 nt sequence GGGGGTGTAG-A-TCAGTGGTAGA is a 5′-tRF from the AlaAGC anticodon (trna117 on chromosome 6). Allowing for exactly one mismatch makes this 5′-tRF indistinguishable from GGGGGTGTAG-C-TCAGTGGTAGA, which appears in 11 isodecoders of three Ala anticodons (AlaAGC, AlaCGC, AlaTGC) as well as in two non-Ala anticodons, namely CysGCA (trna7 on chromosome 3) and ValAAC (trna115 on chromosome 6). Thus, by allowing a single replacement during mapping, reads that arise from any one of these 14 genomic locations would be indistinguishable leading to cross-talk and consequent erroneous estimates about the abundance of 5′-tRFs arising from those tRNAs. To avoid such confounding events we do not permit indels or replacements in our analysis.

### Mapping on tRNA space alone will generate erroneous results

It is tempting to consider compiling a database of tRNA sequences (e.g. by combining all the spliced nuclear and mitochondrial tRNA sequences) and then map the sequenced reads on this subset of the genomic real estate. Such an approach would be easy to implement, fast to execute, and seemingly adequate. Unfortunately, this approach is error-prone and will lead to misrepresentation of expression and miscalculation of relative abundances of the various tRNA anticodons. Indeed, in addition to the multiple instances of *bona fide* nuclear tRNAs, the human genome is also riddled with many instances of nuclear and mitochondrial *tRNA-lookalikes* [35] as well as *partial* tRNA sequences. Thus, any and all reads that simultaneously land inside and outside “tRNA space” (see Methods) must be excluded from consideration since their tRNA provenance cannot be guaranteed. To achieve this objective all sequenced reads must be mapped on the *entire* genome. We illustrate this statement using as an example the 24 nt sequence GCTCCAGTGGCGCAATCGGTTAGC. The sequence is a 5′-tRF of the IleTAT anticodon and appears identically at five genomic instances of this tRNA. However, this sequence also appears outside tRNA space on the forward strand of chromosome 7 between locations 44465584 and 44465607 inclusive (GCh37). This sequence forms part of the 38 nt sequence GCTCCAGTGGCGCAATCGGTTAGCATGCGGTACTTATA that spans locations 44465584 through 44465621. Even though this 38-mer is labeled as a “tRNA” by RepeatMasker it is much shorter than the 93 nt of the typical IleTAT and thus not a *bona fide* tRNA: any reads with a sequence of GCTCCAGTGGCGCAATCGGTTAGC would need to be discarded as their provenance is obviously ambiguous. Consequently, we map the sequenced reads on the whole genome and discard all those reads that land simultaneously inside and outside tRNA space.

Another important consideration here relates to the nontemplated addition of the trinucleotide “CCA” to the 3′ end of mature tRNAs. Since the post-transcriptionally added CCA-tail is absent from the genome's tRNA loci explicit provisions must be made in order to map such reads or they will be inadvertently excluded from consideration. At the same time, and depending on its length, a *CCA-ending* read can exist elsewhere on the genome and outside tRNA space. In such an event, the tRNA provenance of the *CCA-ending* read cannot be guaranteed and it too needs to be excluded from further consideration.

Finally, we note that across all *bona fide* tRNA loci, and accounting for the CCA addition, there exist 505,295 distinct tRNA fragment sequences solely in tRNA space with lengths between 16 and 50 nt inclusive (Supplementary Table S1).

### It is necessary to carry out exact *multi-mapping*

Typical pipelines that map deep-sequencing datasets report reads that can be mapped either unambiguously to a single location (“unique-mapping”) or to a small number of genomic locations. However, considering that the typical tRNA anticodon has multiple genomic instances, neither of these two choices is appropriate. As an example we note that the 72 nt AspGTC sequence TCCTCGTTAGTATAGTGGTGAGTATCCCCGCCTGTCACGCGGGAGACCGGGGTTCGATTCCCCGACGGGGAG appears identically at 11 genomic loci: five on chromosome 1, two on chromosome 6, three on chromosome 12 and one on chromosome 17. Since we work with short RNA-seq profiles, the typical read will be shorter than 72 nt, which will in turn increase the chance that a read is present at multiple genomic locations some of which may not even be related to tRNAs. The multiple instances of tRNA anticodons and the existence of repeating elements like pyknons [37] require that we carry out “exact multi-mapping” (i.e. no indels, no replacements) where we permit a read to map to practically as many locations as possible – see Methods. We then post-process the resulting maps and only keep reads all of whose instances are within tRNA space. For reads that map to multiple locations yet reside exclusively *inside tRNA space* we keep the read counts from only one such genomic locus to avoid multiple counting.

### TRNA fragments arise from three distinct regions of the mature tRNA span

The first analyzed dataset comprises the short-RNA sequencing profiles of lymphoblastoid cell lines (LCLs) from 452 men and women belonging to five different populations: Utah residents with Northern- and Western-European ancestry (CEU), Finnish (FIN), British (GBR), Toscani Italians (TSI) and Yoruba Africans from the city of Ibadan (YRI) [33]. The second dataset comprises 17 normal and 294 breast cancer samples covering the basic hormone profiles from The Cancer Genome Atlas (TCGA) repository at the National Institutes of Health (NIH) [34]. After filtering out tRNA fragments with low support, we were left with 1,573 tRNA fragments in the LCL set and 437 in the BRCA set. In what follows, we will use LCL to refer both to the analyzed 452 individuals and the corresponding collection of 1,573 tRNA fragments. Analogously, we will use BRCA to refer both to the analyzed 311 primary datasets and the corresponding collection of 437 tRNA fragments. We have included the filtered datasets in Supplementary Tables S2 and S3. We should note here that it is likely that in both of these datasets the quantity of tRNA fragments is lower than the actual abundance in the biological sample, due to the possibility that some fragments do not have a phosphate group at the 5′ end or have a phosphate group at the 3′ end. This is a known limitation of the field of tRNA fragment biology at large [21, 38] and is not unique to our analysis.

We categorized each of the fragments based on the specifics of its endpoints within the span of the corresponding mature tRNA and discovered evidence for the following three categories of tRNA fragments: a) fragments whose 5′ terminus begins exactly at the 1^st^ nucleotide of the corresponding mature tRNA (“+1” fragments or 5′-tRFs); b) fragments that are strictly internal to the mature tRNA sequence, i.e. whose 5′ terminus begins at the 2^nd^ nucleotide or further to the right and whose 3′ terminus ends to the left of the first “C” of the nontemplated “CCA” addition to the mature tRNA (“internal” fragments or i-tRFs); and, c) fragments whose 3′ terminus coincides with any of the bases of the “CCA“ terminal addition (“CCA-ending” fragments or 3′-tRFs). The categories of fragments starting at position +1, and the ones within the CCA tail have been described previously and are known as 5′-tRFs and 3′-tRFs, respectively. However, although there were signs of the presence of i-tRFs in early studies [15, 18] they have not been recognized and defined until now as a distinct and rich category of abundant tRFs, in either cell lines or in human tissues. Supplementary Figure S1 shows a schematic presentation of the tRFs mapped on one tRNA instance. Below, we present multi-faceted evidence that i-tRFs are distinct from both 5′-tRFs and 3′-tRFs and, thus, represent a new category of tRNA-derived fragments.

### The i-tRFs have atypical lengths that are characteristic of the internal region and distinct from those of 5′-tRFs and 3′-tRFs

**Figure 1:**
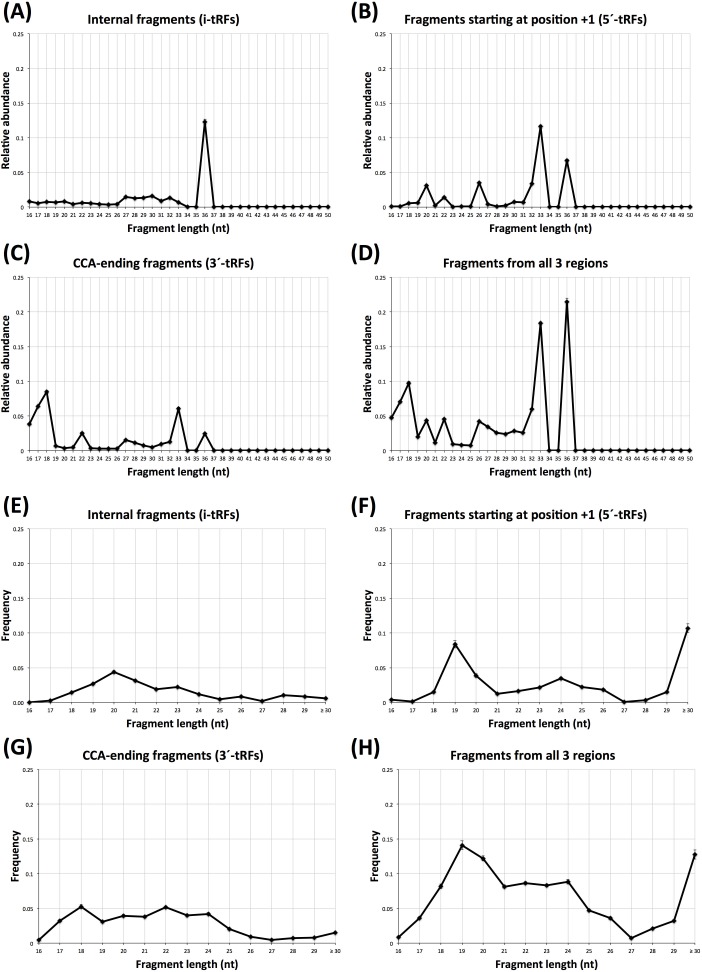
Atypical tRNA fragment lengths Fragment lengths in the 452 individuals of the LCL dataset (**A-D**) and the 311 breast samples (**E**–**H**). **A** and **E**: the length distribution for “internal” fragments only. **B** and **F**: the length distribution for 5′-tRFs only. **C** and **G**: the length distribution for 3′-tRFs. **D** and **H**: the length distribution for all fragments combined. See also text for a detailed explanation of these three shown regions. Error bars are present but barely visible in this Figure and capture standard error across the 452 individuals (A-D) and across the 311 breast samples (E-H). Note the rightmost label of the X-axis in **E-H**: we opted for this label in order to indicate that the observed 30-mers are likely truncated versions of longer-length fragments.

We histogramed the lengths of the reads mapping to the *internal* region and compared them to those of the two known categories of tRNA fragments (5′-tRFs and 3′-tRFs). Figures 1A–1D show the length distributions for the 452 individuals of the LCL dataset. As can be seen from Figure 1A, in these datasets i-tRFs are dominated by a single length, namely 36 nt. By our definition, the 5′ terminus of i-tRFs begins at position +2 of the mature tRNA, or further to the right: consequently, the *internal* 36-mers in LCL comprise the full anticodon triplet (typically centered at position +34 of the mature tRNA sequence) and thus they straddle the point that has been typically associated with the terminus of tRNA halves. Figures 1B and 1C show the length distributions for the *+1* and the *CCA-ending* regions. In Figure 1D we show the combined length distribution. As is evident, each of the three tRNA regions gives rise to fragments with characteristic length profiles and specific relative abundances. Importantly, the very small standard errors (too small to be visible in the four panels) indicate that the lengths of these fragments persist across each of the three regions and across the 452 individuals and thus are not random degradation products.

In the tRNA literature, the 5′-tRFs have been associated with lengths of 18, 22, and 32 nt. In addition to identifying fragments with these lengths, our analysis of the LCL datasets revealed a prevalence for fragments with lengths of 20, 26, 33 and 36 nt: we are not aware of these lengths having been associated previously with 5′-tRFs. Similarly, in LCL, the CCA-ending fragments (3′-tRFs) show prevalence for lengths of 18, 22, 33 and 36 nt. More than half of these 33-mers and 36-mers start *after* the anticodon, which makes many of these fragments distinct from the typical tRNA-halves and thus complementary to the previously reported length-families of 3′-tRFs. It is also worth noting that all the 3′-tRF 33-mers and more than half of the 3′-tRF 36-mers (26 out of 43) originate in mitochondrial tRNA genes.

We repeated the same analysis for the 311 TCGA BRCA datasets. Figure 1E–1H shows the corresponding length distributions. Note how different the i-tRF distribution (Figure 1E) is from those of the 5′-tRFs (Figure 1F) and the 3′-tRFs (Figure 1G): i-tRFs comprise a lot of fragments that are 20 nt long and virtually no fragments ≥30 nt, whereas the 5′-tRFs are characterized by a prevalence of fragments with lengths 19 and ≥30 nt. Just like the case of LCLs, the lengths of the fragments arising from each of the three regions have characteristic profiles and specific relative abundances. Moreover, the very small standard errors (barely visible in the Figure) indicate that the atypical lengths of these fragments remain consistent across the analyzed datasets. It is important to emphasize that these NIH-TCGA datasets were obtained by running the deep sequencing PCR for a total of 30 sequencing cycles. Consequently, any short fragment that may exist in each sample's milieu and is longer than 30 nt [39], will be represented by a 30-mer “proxy.” Also, a considerable portion of the *CCA-ending* fragments in the BRCA datasets have lengths that have not been previously associated with 3′-tRFs [40]. In all, these datasets revealed several length families that have not been previously reported: these families comprise fragments with lengths of 16, 20, 21, and 23-29 nt and collectively account for 21.2% of the 3′-tRFs in the BRCA datasets. Lastly, we note that we will delve further into the significance of the length distribution of i-tRFs in breast tissue in the following analyses.

### i-tRFs represent a diverse new family of tRNA fragments

**Figure 2:**
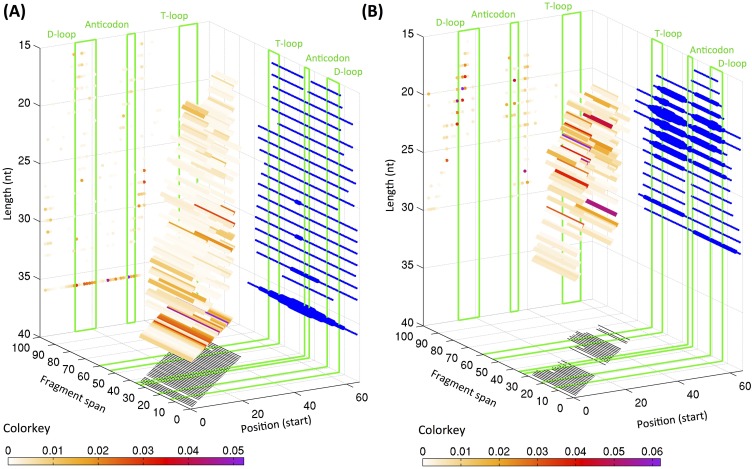
Distribution of starting position and lengths for i-tRFs 3D graphs showing the starting positions of the internal tRNA fragments, their span and lengths in the LCL (**A**) and BRCA (**B**) datasets. The positions are numbered with reference to the +1 position of the mature tRNA. The representative positions for the D- and T-loops as well as for the anticodon loop are highlighted with green boxes. The coloring of each bar is proportional to the relative abundance of each length of the fragments starting at that specific position as indicated by the respective color-key below each graph. The thickness of the projections on the right wall of the graph is proportional to the number of fragments spanning the specific position. For the LCL dataset, only the top 50% most expressed internal fragments are shown.

Our analyses revealed i*–*tRFs to be a surprisingly rich category with many of its members having 5′ termini that are away from the 5′ end of the mature tRNA: i-tRFs represent 27.5% of all fragments in the LCL and 21.0% of all fragments in the BRCA dataset. Figure 2 shows the distribution of the starting positions of the i-tRFs for the LCL (panel A) and BRCA datasets (panel B): for each starting position, the length distribution is also shown as colored bars, with the color of each bar representing the average expression of the respective fragment in the LCL or BRCA dataset. For the LCL dataset, *internal* 36-mers can begin anywhere within the D-loop of the mature tRNA (generally positions 12-22) or immediately after it (in 5′ → 3′ orientation). As is evident, no specific position can be singled out as the *preferred* starting position of internal fragments in this dataset (Figure 2A). On the contrary, in the BRCA dataset, there are two main “clusters” of starting positions for the i-tRFs: a first cluster spanning positions 11-17 that generally reside in the D-loop and a second cluster spanning positions 32-43 comprise the anticodon loop and the variable loop of the mature tRNA. Importantly, each of the starting positions exhibited its own associated range of lengths for the fragments that began there: fragments that began at position 13 were 23, 22 or 21 nt long whereas fragments that began at position 15 or 16 were slightly shorter with lengths 19, 20, or 21 nt. As we have already seen that these fragment lengths recur in both the LCL and BRCA datasets and have very small standard deviations (Figure 1), we surmise that the mechanisms behind the production of these fragments have specific preferences for the starting and ending positions and/or the length of the tRNA fragment. We conjecture that some of the molecules that appear to be 30-mers in Figure 2B starting at positions 2, 34 and 36 might be tRNA-halves that cannot be “seen” as such due to the 30-PCR-cycle limitation in the breast datasets that we mentioned above.

### TRFs from all three regions exhibit diversities and abundances that depend strongly on the choice of anticodon and differ between nuclearly- and mitochondrially-encoded tRNAs

For each of the two collections of analyzed datasets, and separately for each anticodon, we enumerated the fragments arising from all of the *bona fide* genomic instances of the anticodon being considered each time. In each case, we also determined how many fragments arise from which of the three regions of the mature tRNA, namely “*+1*”, “*internal*,” or “*CCA-ending*.” The results are summarized in Supplementary Table S4 (LCL datasets) and Supplementary Table S5 (BRCA datasets). We also enumerated the fragments originating from *pseudo*-tRNAs and from sequences of potential *pseudo*-tRNA origin and found them to be considerably fewer than those from true tRNAs (Supplementary Table S6).

In the LCL collection, we find 63 anticodons (from a possible total of 75 nuclear and mitochondrial ones) that generate fragments with abundance levels that meet our mapping and filtering criteria. The *mitochondrial* tRNA GluTTC generates the highest number of distinct tRNA fragments followed by the *nuclear* LysCTT. Notably, we found that the diversity of fragments that arise from each of the three regions of the mature tRNA strongly depends on the anticodon at hand (Supplementary Table S4). Indeed, for some anticodons the “*+1*” region gives rise to the most diverse set of tRNA fragments (e.g. nuclear GluTTC) whereas for other anticodons most of the diversity is encountered in the *internal* (e.g. mitochondrial HisGTG) or the *CCA-ending* region (e.g. mitochondrial ValTAC).

Analogously, in the BRCA collection, we find that 52 of the 75 possible nuclear and mitochondrial anticodons generate fragments satisfying our filtering criteria. As with the LCL datasets, here too the diversity of fragments that arise from each of the three regions of the mature tRNA strongly depends on the considered anticodon (Supplementary Table S5). Similarly to the LCL collection, the mitochondrial GluTTC produces the highest number of distinct fragments here as well whereas the mitochondrial ValTAC gives rise mainly to *CCA-ending* fragments.

The analysis of these two different types of datasets also revealed examples of anticodons where the fragment profile changes with the tissue type (see also below). For example, in the LCL datasets, the nuclear AlaACG generates predominantly *CCA-ending* fragments. On the other hand, in the BRCA datasets the anticodon's 5′-tRFs are favored as well and are produced at a ratio of 1:1 compared to the 3′-tRFs. Additional details can be found in Supplementary Tables S4 and S5.

Additionally, we found that the *abundance* of the tRNA fragments exhibits anticodon-dependencies as well. In fact, from this standpoint the differences between the LCL and the BRCA collections are more pronounced. In the LCL dataset, the relative abundances of different fragment lengths are due to fragments from different anticodons. As shown in Supplementary Figure S2, the mitochondrial SerGTC anticodon is responsible for 68.7% and 80.4% of the contribution to fragments with the previously unreported lengths of 20 and 26 nt. On the other hand, for fragments of length 36 nt, it is the nuclear GluCTC, nuclear GluTTC, and the mitochondrial GluTTC anticodons that account for 37.9% of all 36-mers, with the rest being contributed by an assortment of anticodons. Interestingly, in the BRCA datasets, the mitochondrial ValTAC anticodon generates approximately 30.0% of the fragments with lengths of 20-23 nt (Supplementary Figure S3).

We further sought to examine the relationship between tRNA fragment lengths and abundances, and their genomic origin (i.e. whether nuclearly-encoded *vs*. mitochondrially-encoded). To this end, we decomposed the graphs of Figures 1D and 1H into their nuclear and the mitochondrial contributions (Supplementary Figure S4). We identified several statistically significant differences in the expression of nuclear and mitochondrial tRNAs in both the LCL and the BRCA dataset (Supplementary Figure S4). Notably, the 36-mers in the LCL dataset are predominantly from mitochondrially-encoded tRNAs, while the 33-mers are from nuclearly-encoded ones. This result, combined with the observations from Figure 1 on the region-dependencies of each length, suggests links between the length of a fragment, its DNA origin, and which of the three tRNA regions it is part of.

### TRFs from the same anticodon have poorly-correlated abundances

**Figure 3:**
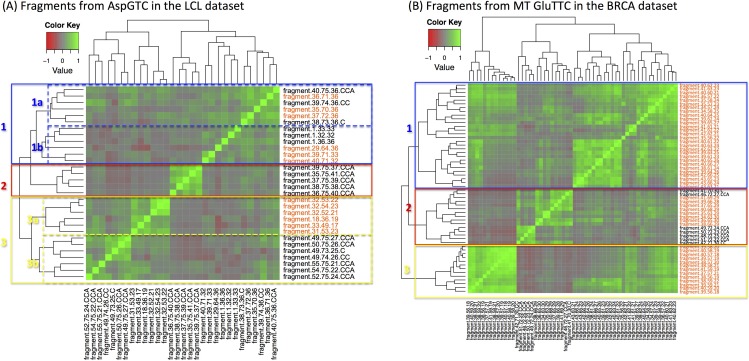
Uncorrelated abundances Heatmap of the Pearson correlation coefficient for statistically significant fragments. **A:** Case of tRNA fragments that arise from the nuclear AspGTC (trna10 on chromosome 12) anticodon in the LCL dataset. **B:** Case of tRNA fragments, mostly i-tRFs, which arise from the mitochondrial GluTTC anticodon in the BRCA dataset. Several mini-clusters are evident in each heatmap: however, there is correlation across the mini-clusters of the same tRNA (see text for a detailed explanation). Orange-colored labels mark the i-tRFs.

Considering the richness of fragments that can arise from a given anticodon (Supplementary Tables S4 and S5), we investigated whether their abundances are correlated. Figure 3A shows a Pearson correlation heatmap for the fragments arising from the *nuclear* AspGTC in the LCL datasets. Figure 3B shows the analogous heatmap for the *mitochondrial* GluTTC in the BRCA datasets: as we saw above, this anticodon produces the largest number of fragments in the BRCA datasets and most of them are *internal*, i.e. i-tRFs. The abundances of reads originating from the three tRNA regions (i.e., “*+1*,” “*internal*,” “*CCA-ending*”) show a poor correlation. Similarly poor correlation characterizes fragments that arise from the same anticodon yet are of different lengths. We note however the existence of several small clusters in these heatmaps. For the nuclear AspGTC (LCL datasets – Figure 3A), cluster 1a comprises *internal* and *CCA-ending* 36-mers whereas cluster 1b captures mainly *internal* 32-mers and 33-mers. Cluster 2 comprises *CCA-ending* fragments that are 37 nt or longer. Cluster 3b contains *CCA-ending* fragments between 24 and 27 nt whereas cluster 3c comprises *internal* fragments between 17 and 23 nt. Analogous observations can be made for the fragments for the mitochondrial GluTTC fragments (BRCA datasets – Figure 3B): short *internal* fragments, generally of length 21 nt or shorter, form cluster 3, while *internal* fragments of intermediate length (21-27 nt) comprise cluster 1. On the other hand, cluster 2 contains long *internal* fragments and all of the *CCA-ending* fragments from this anticodon. A mini sub-cluster of cluster 2 comprising the shorter *CCA-ending* fragments (22-25 nt) is also evident.

Examination of the Pearson correlation maps for the other anticodons shows that they are qualitatively similar to the ones shown in the two panels of Figure 3. This allows us to make two general observations. First, we find evidence in all anticodons for well-defined mini-clusters each of which contains only a few of the anticodon's fragments: the members of each such mini-cluster have correlated abundances. Second, when we compare a given anticodon's mini-clusters with one another, we observe a characteristic *absence of correlation* even in cases where fragments from two mini-clusters overlap on the mature tRNA sequence from which they originate (see, for example, the mini-clusters 1a and 2 in Figure 3A, or clusters 1 and 3 in Figure 3B). These observations, in conjunction with the very small standard errors across the 452 (LCL) and 311 (BRCA) individuals shown in Figure 1, give more weight to the view that the fragments from all three regions of the mature tRNA are constitutive in nature and not random degradation products.

### TRFs have lengths that depend on tissue type and tissue state

Inspection of the distributions shown in Figures 1 and 2 indicates that the specifics of 5′-tRFs, i-tRFs, and 3′-tRFs depend strongly on the tissue. Looking at the BRCA datasets (and without distinguishing between the normal and tumor datasets), it is evident that the dominant fragments here have lengths between 19 and 24 nt and account for 60.2% of all tRNA fragments in this collection. By contrast, in the LCL datasets, the dominant fragments have lengths of 18, 33, and 36 nt and account for nearly 50% of all tRFs.

**Figure 4:**
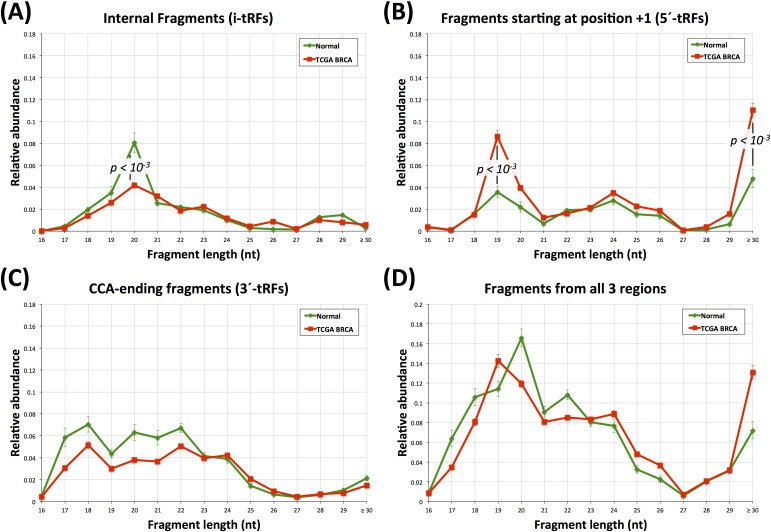
Fragment lengths in the breast datasets Atypical tRF lengths in normal and tumor breast datasets. **A**: length distributions for the i-tRFs. **B**: length distributions for 5′-tRFs only. **C**: length distribution for the 3′-tRFs. **D**: length distribution for all the fragments combined. Green curve: normal dataset fragments. Red curve: tumor dataset fragments. For the 19-mer and 30-mer 5′-tRFs as well as for the 20-mer i-tRFs the statistical significance (Mann-Witney U-test; *p*-val < 10^−3^) between the normal and the tumor datasets is indicated. Error bars capture standard error across the analyzed groups of datasets.

To increase our resolving power we further decomposed the BRCA fragment distributions of Figure 1E–1H into their two constituent parts, namely the subset of normal datasets and that of the tumor datasets (Figure 4).

The tissue-type differences that exist between the normal BRCA and the normal LCL datasets are now more evident. In the *internal* region, 36-mers i-tRFs take the lion's share in the LCL set (Figure 1A) whereas in the BRCA set 20-mer i-tRFs provide a modest contribution to the total pool of fragments in the normal breast datasets (Figure 4A). In the *+1* region, 5′-tRFs with length 19 nt (Figure 4B) are the dominant population in normal breast (compare this with the 33-mers and 36-mers in the *+1* region in LCLs shown in Figure 1B). Lastly, the *CCA-ending* region is dominated by 17-mer, 18-mer and 33-mer 3′-tRFs in LCL (Figure 1C) yet shows a fairly uniform distribution in i-tRFs with lengths 17-24 nt in normal breast (Figure 4C).

Having decomposed the BRCA distribution into its normal (17 datasets) and tumor (294) components we next sought to identify similarities and differences that might depend on tissue state. We find that the most striking differences are among the i′-tRFs and the 5′-tRFs, which suggests an intriguing and currently unexplored interconnection between the two categories of fragments. As can be seen from Figure 4, the the proportion of *internal* fragments with length 20 nt is nearly halved in the tumor datasets compared to normal (*p*-val < 10^−3^) whereas the proportion of 5′-tRFs with length 19 nt and with lengths ≥ 30 nt more than doubles in the tumor datasets (*p*-val < 10^−3^ for both comparisons). It appears as if the normal datasets preferentially produce i-tRFs while also reducing the expression of the 5′-tRFs, with a reversal of this situation occurring in the tumor. Notably, the relative abundance for the rest of the i-tRFs and 5′-tRFs remains largely unchanged between normal and tumor.

### TRFs have relative abundances that are tissue-specific and tissue-state-specific

In the context of messenger RNA (mRNA) expression studies, the abundance profiles of mRNAs that are common to two tissues can be used to tell the tissues apart (tissue-specific mRNA “signatures”). Similarly, for a given tissue, mRNA abundance profiles can distinguish between normal and disease states (tissue-state-specific mRNA “signatures”). Naturally, we wondered whether tRFs possess similar properties.

**Figure 5:**
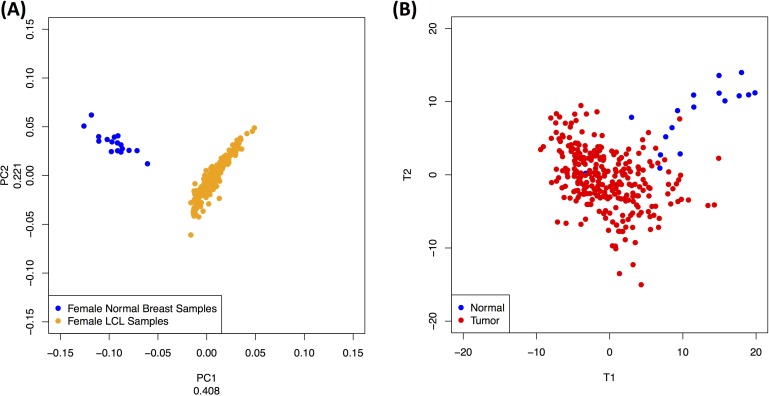
Dependence on tissue and tissue-state Looking at tRFs that are present in two tissues we find that they have tissue- and tissue-state specific abundances. **A**: PCA (unsupervised) of the abundance levels of the 200 tRNA fragments that are common to female LCL datasets and to the normal breast datasets can distinguish between the two tissues. **B**: PLS-DA (supervised) of the abundance levels of the 437 tRFs found in the BRCA dataset can distinguish between the two groups. See also text.

To investigate the possibility of a tissue-specific profile, we focused solely on the 200 tRFs that are common to the following two datasets: a) the subset of 253 female datasets from the LCL dataset (all of whom are healthy), and b) the 17 normal (female) datasets from the BRCA dataset. In Figure 5A, we show that a principal component analysis (unsupervised) of the abundances of these 200 fragments can easily distinguish between the two tissues. It is important to note how characteristically tight each of the two point clusters is: this indicates that the abundance profiles of these 200 tRNA fragments are very similar across all datasets belonging to the same cluster. We note that this evident within-group similarity of the tRF abundance profiles further supports the view that these fragments are constitutive in nature and not degradation products.

As the LCL and BRCA datasets come from two distinct studies, we wanted to exclude the possibility that the differences we see are due to biases caused by either the sequencing methods and/or by the whole experimental handling of the datasets [41, 42]. Due to the lack of standard datasets that were common to both studies, we truncated the data by rank-normalizing the two datasets. By ranking the expression in each dataset, much of the quantitative information is lost and only the relative ordering based on abundance is retained [43]. By performing PCA on this truncated dataset we can still distinguish easily the two datasets (Supplementary Figure S5), which indicates that the differences in the abundance profiles have a biological basis and are not due to experimental biases. We also used SAM [44], a non-parametric significance analysis method, to identify quantitative differences between the two datasets. We found that most of the fragments are differentially abundant between the two tissues (Supplementary Figure S5B). More than 30% of the significantly differentiated fragments are in fact i-tRFs (Supplementary Table S7), which further argues for the importance of this novel category of tRFs.

To investigate the possibility of a tissue-state-specific profile, we formed a single group by combining all tumor datasets independently of hormone status. Unlike the above example, we are now dealing with an artificially increased underlying heterogeneity, the result of our having combined all breast cancer subtypes into a single group of datasets. We thus opted for a *supervised* clustering approach, namely PLS-DA [45]. Figure 5B shows that PLS-DA can easily distinguish between the two sets based on the abundance levels of these tRFs. It is also worth pointing out how the tumor dataset heterogeneity is reflected by the lack of tightness in the formed tumor cluster of Figure 5B.

### TRFs and in particular i-tRFs exhibit race-dependent differences at the molecular, cellular and tissue levels

In recent work, we reported on transcripts whose abundance profiles differ across human races [46], between males and females of the same population [47], and between population groups [47]. Considering that both the LCL and the BRCA datasets include individuals belonging to different races, we sought to determine whether the abundance profiles of the tRFs exhibited any differences along this dimension.

**Figure 6:**
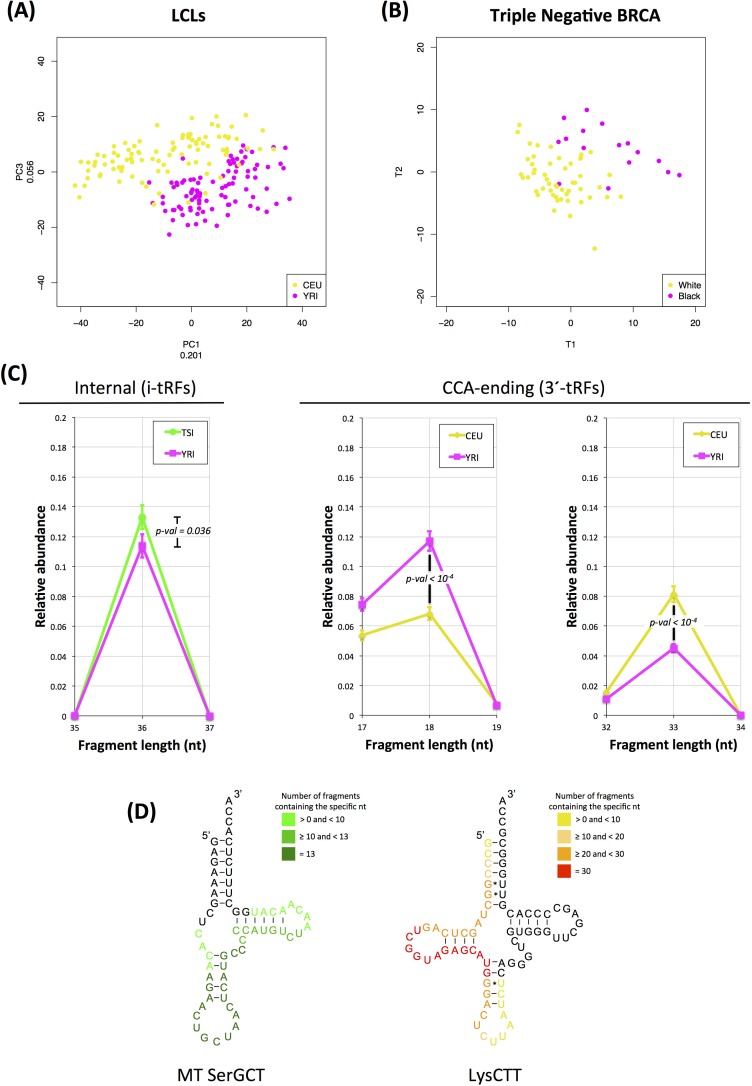
Dependence on race Race-dependent abundance profiles for statistically significant tRNA fragments. **A**: Principal components analysis of fragment expression in LCLs. The CEU population (white) is represented by the yellow points whereas the YRI population (black) is represented by the magenta points. Both men and women from the two populations were included in this analysis. The number next to the label of each axis indicates the amount of variance that the corresponding principal component explains. **B**: Partial Least Squares – Discriminant Analysis on the tRNA fragments in the 78 triple-negative-breast-cancer datasets. The yellow points represent white patients where the magenta dots represent black patients. See also text for details. **C**: Relative abundances of 36-mer i-tRFs for the FIN and YRI populations (left panel) and 18-mer 3′-tRFs (middle panel) and 33-mer 3′-tRFs (right panel) for the CEU and YRI datasets. The differences for all three comparisons are statistically significant as indicated by the respective p-value on each graph (Mann-Whitney U-test). Error bars capture the standard error of the relative abundance of each type of fragments for *n* = 93 (CEU) and *n* = 95 (YRI) datasets. **D**: Map of the nucleotides of differentially expressed fragments between the YRI and the CEU populations as projected on the respective mature tRNA. Each base is colored based on the number of distinct fragments containing it. As reference for the LysCTT, the trna10 of this anticodon on chromosome 16 was used. The full list of significantly differentiated fragments between the two populations is included in Supplementary Table S8.

First, we compared the transcriptional profiles in the 93 LCL datasets from the CEU (white, men and women) group *vs*. the 95 datasets from the YRI (black, men and women) group. Figure 6A shows the results of the (*unsupervised*) principal component analysis for these two subsets. As is evident, the 1^st^ and 3^rd^ principal component provide a good separation of the two groups with only modest crosstalk. This indicates that the tRNA fragments exhibit race-dependent transcriptional differences at *the cellular level* (EBV-immortalized B-cells).

Second, we focused on the 78 triple negative breast cancer datasets from the BRCA dataset. This subset comprises an adequate number of black (16) and white (51) patients to permit statistical analyses. Because of the known underlying heterogeneity of this particular subtype, we opted for a *supervised* approach (PLS-DA). As Figure 6B shows, there is an evident separation between the white and black patients that is again characterized by only modest crosstalk. This is analogous to the above result with the LCL datasets and indicates that the tRNA fragments exhibit race-dependent transcriptional differences at *the tissue level* as well.

Third, we investigated the possibility that such differences exist among populations. To this end, we decomposed the graphs of Figure 1A–1D into their constituent components, one for each of the five populations (the full graph for all populations is shown in Supplementary Figure S6). Qualitatively, the length distributions of all five populations follow a similar pattern. However, a closer look reveals statistically significant differences in the distributions of the tRF lengths among races. The left panel of Figure 6C shows that the novel category of i-tRFs contains population-dependent differences: the YRI population is characterized by significantly lower abundance of 36-mer i-tRFs compared to the TSI population. In Figure 6C, two details from the CEU and YRI distributions are also shown on the middle and right panel. Here we see that there are nearly twice as many 18-mer 3′-tRFs in the YRI population compared to the CEU population (*p*-val ≤ 10^−4^). The situation is reversed for 33-mer 3′-tRFs with the CEU population now having twice as many 33-mers as the YRI population (*p*-val ≤ 10^−4^). These quantitative and statistically significant differences in the fragments produced by the CEU and YRI indicate that the tRNA fragments exhibit race-dependent transcriptional differences at *the molecular level* as well.

In light of these observations, we sought to determine which tRNA fragments have significantly different abundances between the CEU and YRI populations in a multivariate way. Using SAM [44], a nonparametric significance analysis method, at a strict FDR setting of 0.00% we identified 93 tRFs with differential abundances: 48 had lower abundance in the YRI datasets compared to the CEU ones whereas the remaining 45 had higher abundance (Supplementary Figure S7 and Table S8). Notably, we found that most of the differentially abundant fragments are i-tRFs, which further highlights the significance of the internal region. The majority of tRNA fragments that exhibit lower expression in the YRI group originate in the mitochondria: specifically, they are i-tRFs from the *internal* region of the mitochondrial SerGCT tRNA that straddle the anticodon (they begin around position +13 and end around position +43) (Figure 6D). In addition to SerGCT, the mitochondrial ValTAC and mitochondrial PheGAA also contribute considerably to the list of fragments that are differentially expressed between CEU and YRI.

Among the fragments that had higher expression in the YRI datasets compared to the CEU ones and were identified by SAM, those originating from the nuclear LysCTT anticodon dominated. Of the 45 tRNA fragments that are significantly significant, 30 arise from the LysCTT template. An additional 5 statistically significant tRNA fragments with higher abundance in the YRI datasets are contributed by the LysTTT anticodon. Only 2 of the 30 statistically significant LysCTT fragments begin at position +1, and, thus, are classic 5′ tRNA-halves). The remaining 28 of the 30 statistically significant LysCTT fragments are i-tRFs, i.e. they arise from the *internal* region: they begin between positions 2 and 7 inclusive of the mature tRNA and end just before the anticodon triplet (position +33 using trna10 of LysCTT on chromosome 13 as a reference, Figure 6D); these i-tRFs have no apparent length consensus (21-33 nt).

### TRFs exhibit gender-dependencies

We also examined the possibility that the tRFs show differences across gender boundaries. As mentioned above, we recently reported on transcripts whose abundance profiles differ between males and females [47], thus making this a possibility worth investigating.

**Figure 7:**
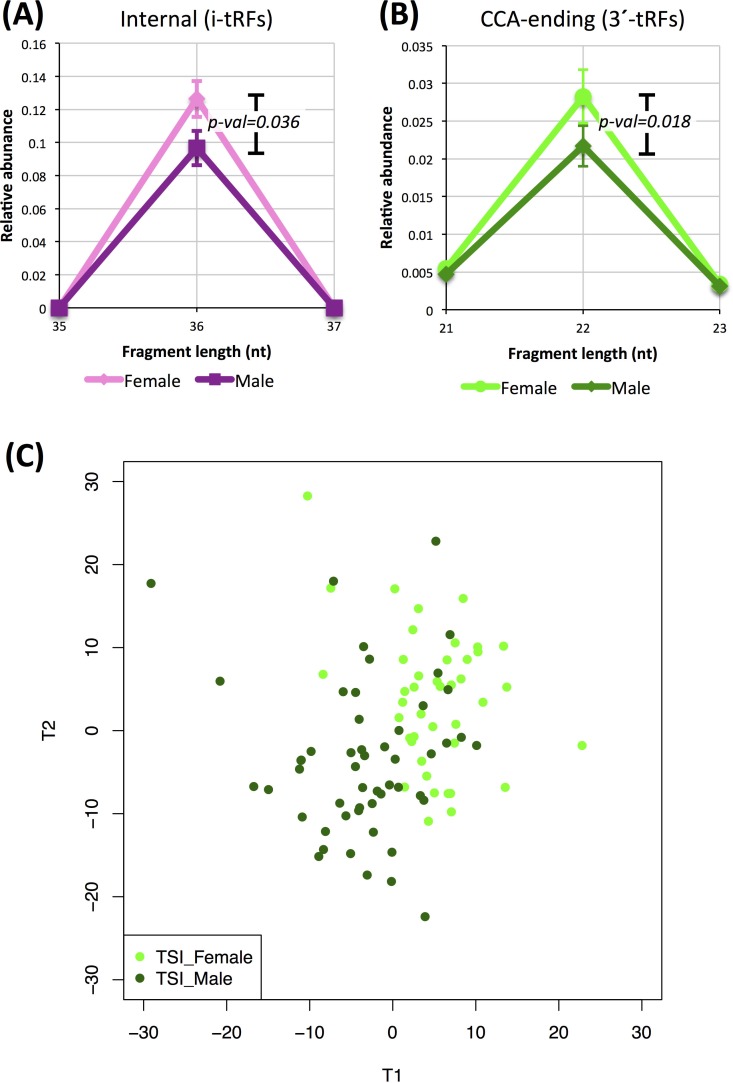
Dependence on gender Differences in the abundance of tRNA fragments between men and women. **A**: Detail from the length distributions for YRI men and women for internal fragments. **B**: Detail from the length distributions for TSI men and women for CCA-ending fragments. The difference in abundance is statistically significant in both comparisons (Mann-Whitney U-test). Error bars in (**A**) and (**B**) capture standard error across the analyzed groups of datasets. **C**: PLS-DA graph of TSI men and TSI women showing a trend for gender-specific tRNA profiles. The important fragments for the projection (VIP score > 1.5) are provided in Supplementary Table S9.

Among the 452 individuals of the LCL dataset, both genders and the five populations (CEU, FIN, GBR, TSI, YRI) are represented evenly. Our analyses indicate that there is a tendency for separation but not a sharp discrimination between the two genders. Focusing on the *internal* fragments, we first decomposed the fragment length distributions of Figure 1A but did so separately for men and women and for the five populations. Figures 7A shows two details of the distributions for men and women (YRI datasets only) for the *internal* 36-mers. These i-tRFs are less abundant in YRI males compared to YRI females (*p*-val = 0.036). Figure 7B shows an analogous detail for *CCA-ending* fragments for men and women (TSI datasets only). In the TSI population these 22-mers are more abundant in women compared to men with the difference been statistically significant (*p*-val = 0.018). Using PLS-DA on the TSI men and women we can discern a trend for separation of the two genders (Figure 7C). Among the fragments that are significant for the construction of the PLS-DA-driven separation (VIP scores > 1.5) more than half (49 out of 94; Supplementary Table S9) are i-tRFs.

### The abundances of tRFs depend on disease subtype

The different tumor subtypes captured by the BRCA datasets we analyzed allowed us to investigate whether the profiles of tRFs differ between tumor subcategories. For this analysis, we focused on three subsets: the normal breast datasets, the ER−/PR−/HER2− (triple negative) datasets, and the ER+/PR+/HER2+ (triple positive) datasets [48]. Since we have already shown that the tRF profiles are race-dependent, we chose to work with a single race and in particular white women who were represented in the BRCA collection at adequately high numbers (15 normal, 24 triple positive and 51 triple negative datasets).

**Figure 8:**
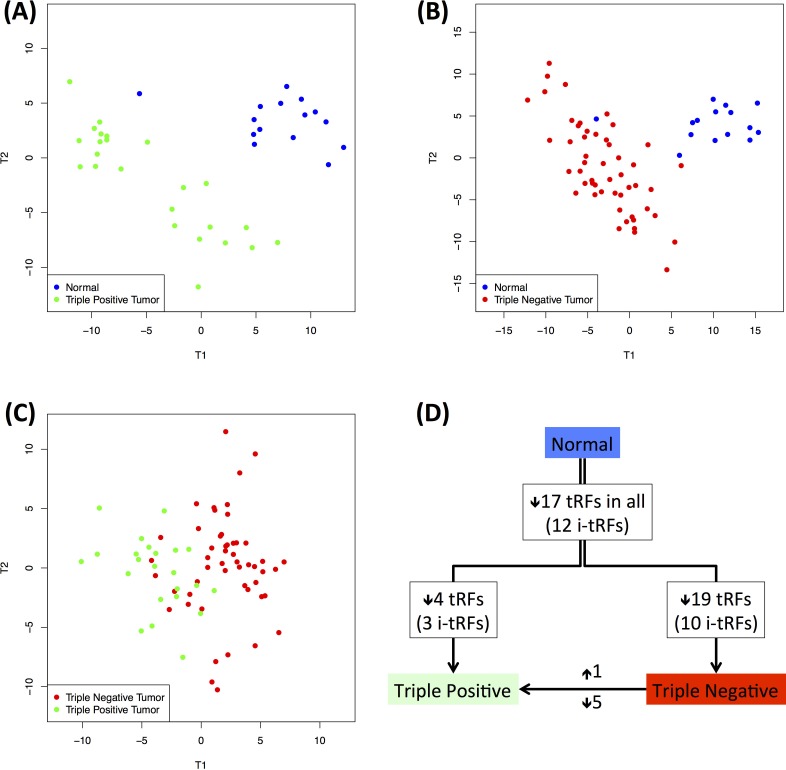
Dependence on disease state Differences in the tRNA profiles between normal and disease states (in white individuals only). **A**: PLS-DA graph for the discrimination of normal and triple positive datasets. **B**: PLS-DA graph for the discrimination of normal and triple negative datasets. **C**: PLS-DA can also discriminate between the two subtypes. **D**: The fragments that are important for each separation were used to identify disease subtype-specific abundance changes. The number of fragments with higher or lower abundance is indicated next to each arrow; the number of i-tRFs in each case is shown parenthesized. Each arrow represents a comparison between two groups: the start of the arrow indicates the “control” group compared to which the fragments in the “target” group (end of arrow) have altered abundance. A detailed list of the fragments is given in Supplementary Table S10.

We performed pair-wise PLS-DA analyses and found that in all three cases, the two subtypes being compared can be discriminated clearly from one another (Figure 8 – panels A, B and C). Importantly, the ability to discriminate the two tumor subtypes based on tRNA fragment abundance suggests a potentially significant role for these fragments in the respective biology of these breast cancer subtypes.

All of the statistically significant tRFs had lower abundance in the tumor datasets compared to the normal datasets (Figure 8D). We cross-validated the findings through an independent SAM analysis (see Methods, Supplementary Table S10, and Figure S7). In concordance with the PLS-DA model, SAM also identified the same 17 fragments as having lower abundance in each tumor subtype compared to the normal datasets. Triple negative tumors were characterized by an *additional* 19 fragments that had lower abundance in the tumor compared to the normal datasets (for a total of 36 fragments in the triple negative subtype). It is important to also note that the majority of differentially abundant tRFs in the two normal *vs*. tumor comparisons are from the *internal* region, i.e. i-tRFs (Figure 8). On the other hand, in the intra-tumor comparison, the differentially abundant tRFs are all 5′-tRFs and most of them are 19-mers from different genomic loci of the nuclear ArgTCG anticodon (Supplementary Table S10). These findings are in concordance with Figures 4A and 4B and were validated by two independent statistical methods (PLS-DA and SAM), which in turn suggests the existence of concrete differences in the abundance of the tRNA fragment population in the two disease subtypes.

### TRFs are loaded on Argonaute in a cell-line-specific manner

Previous work [20, 21, 49] demonstrated that tRFs can be loaded on Argonaute (Ago) which indicates that one of their functions is through the RNAi pathway. We are not aware however of any reported studies that have examined *differential* Ago-loading of tRFs as a function of tissue, tissue-state, race, or disease subtype. To this end, we analyzed the publicly available Ago HITS-CLIP datasets for three different breast cancer cell lines each of which models specific breast cancer categories [50]. For consistency, and since the TCGA-BRCA dataset contained only reads ≤ 30 nt, we analyzed the HITS-CLIP datasets using only fragments ≤ 30 nt long. We included the filtered raw data in Supplementary Table S11. 70 of the abundant fragments originated in the *internal* (i-tRFs) and 68 in the *CCA-ending* (3′-tRFs) regions; by comparison, only 25 abundant 5′-tRFs were loaded on Argonaute.

**Figure 9:**
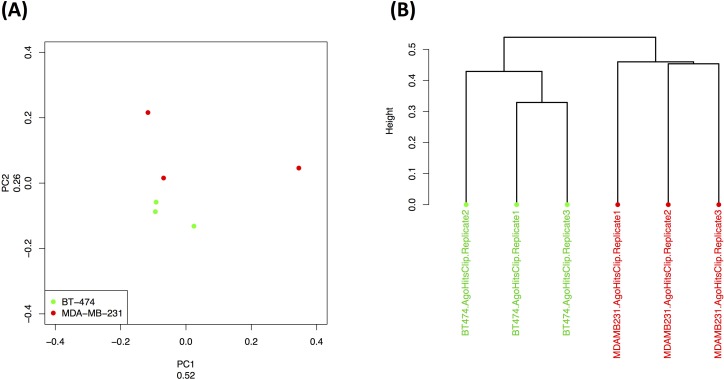
Ago-loading dependence on cell sub-type Cell-line-specific Ago-loaded tRF profiles. Unsupervised PCA (**A**) and Hierarchical Clustering (**B**) discriminated the replicates of two model cell lines for triple negative (MDA-MB-231) and triple positive (BT-474) breast tumors. Kendall's tau coefficient was used as distance metric for the dendrogram in (**B**).

As we demonstrated above, the triple negative and triple positive breast tumor subtypes can be discriminated based on the *transcriptional* abundance profile of their tRFs. Thus, we first examined if the Ago-loaded tRNA fragments can also distinguish between these two tumor subtypes: as shown in Figure 9A, unsupervised PCA can easily separate the BT-474 (triple positive) and MBA-231 (triple negative) cell line data (three replicates each). This is further corroborated by the independent clustering of the same data by Hierarchical Clustering (Figure 9B). This is an important and interesting result: what drives this separation is not the presence or absence of specific tRFs but rather their *differential Ago-loading* in each cell line. Indeed, we refer the reader to the Venn diagram of Supplementary Figure S9A where we examine the overlap among the tRF populations in all three cell lines [50]: the Venn diagram shows that the vast majority of the Ago-loaded tRFs are present in all three cell lines whereas there are only two out of the 163 tRFs that are unique to only one cell line.

Additionally we examined the length distributions of all Ago-loaded tRFs with length ≤ 30 nt in the three cell lines. Interestingly, we found that each cell line has its own distinct profile of Ago-loaded fragments (Supplementary Figure S9B-E). In particular, BT-474 cells exhibited a peak for 26-mers that is due mainly to i-tRFs. On the other hand, MDA-MB-231 had a prevalence for Ago-loaded 16-mers, 17-mers, and 21-mers 3′-tRFs and for 23-mers that are of *internal* origin (i-tRFs). In MCF-7, we also noted a “gradient” in the length of Ago-loaded fragments per tRNA region. The *+1* region contributed the longest (28-30 nt) among the Ago-loaded fragments whereas the *CCA-ending* region contributed the shortest (16-18 nt) among them. In MCF-7, the *internal* region was represented by Ago-loaded tRNA fragments with intermediate lengths (21-23 nt). SAM analysis identified seven fragments that are loaded in statistically significantly fewer amounts on Ago in MDA-MB-231 compared to BT-474 (Supplementary Figure S9F and Supplementary Table S12): six of these seven differentially expressed fragments are i-tRFs and from two specific anticodons: the mitochondrial ProTGG and the nuclear HisGTG. These findings support a model where the tRNA fragments are preferentially Ago-loaded in a manner that is cell-line-specific, presumably reflecting disease-subtype specificity. The findings also corroborate recently proposed functional roles for the shorter among the tRFs [20, 21] through their participation in the RNAi pathway as miRNA-like entities.

### Fragment-specific PCR-based validation of expression of *internal* tRNA fragments

**Figure 10:**
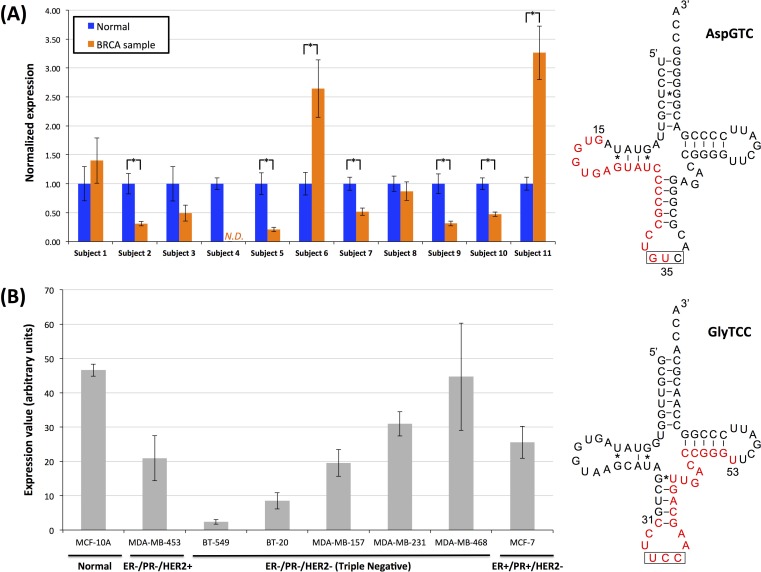
Internal fragments in breast samples and breast cell lines Experimental validation of two internal fragments. **A**: Quantification of the i-tRF from the nuclear AspGTC anticodon in 11 breast tumor and 11 adjacent normal breast samples. N.D.: not determined; in this case, the fragment's expression was too low to be detected. Stars indicate statistically significant changes in abundance (*p*-val < 0.01; Student's *t*-test) between the tumor and adjacent normal tissue of the same subject. In all cases there were n = 3 repetitions of the experiments. Error bars show the standard deviation. **B**: Quantification of the i-tRF from the nuclear GlyTCC anticodon in eight different normal and breast cancer cell lines. Column height represents the average expression value and error bars the standard deviation of at least 10 independent measurements in each sample. On the right hand-side of (**A**) and (**B**), the examined fragment is highlighted in red. The anticodon triplet is highlighted by the black box. The genomic coordinates of the depicted AspGTC tRNA are from 125424264 to 125424193, inclusive, on chromosome 12, while for the depicted GlyTCC tRNA are from 8124866 to 8124937, inclusive, on chromosome 17. ER: Estrogen Receptor status, PR: Progesterone Receptor status, HER2: Human Epidermal Growth Factor Receptor 2 status.

As the tRFs that arise from the *internal* region of mature tRNAs represent a novel category of tRFs we sought independent experimental validation for these novel molecules. For this purpose, we selected one i-tRF that begins within the loop region of the D-loop of AspGTC and ends at the anticodon (Figure 10A) and one that starts before the anticodon loop and ends at the T-loop of GlyTCC (Figure 10B); both fragments were identified repeatedly in our analyses of the BRCA datasets. What makes the quantification task challenging is the requirement to amplify the fragment while also specifically ensuring that the amplified molecule has exactly the endpoints captured by the RNA-seq datasets. To this end, we devised a novel PCR method [51] that is able to detect only RNA molecules with specified length and with specified endpoints; the method relies on the ligation of specific adaptors followed by TaqMan PCR quantification (Supplementary Figure S8; see Methods). We also exploited the Multiplex miRNA Assay [52, 53], a method with single nucleotide specificity, for quantification of the second tRF. The starting material for our experiments was total RNA extracted from 11 breast tumor and from 11 adjacent normal breast samples (Supplementary Table S13 lists the hormone profile of these samples, the pathology diagnosis etc.) for the quantification of the fragment from the AspGTC tRNA and total RNA from eight different normal or breast cancer cell lines for the quantification of the GlyTCC-derived fragment.

Each tissue RNA sample was subjected to ligation with specific adaptors for the tRF from the AspGTC anticodon (Supplementary Table S14; Supplementary Figure S8) and to q-PCR quantification as outlined in Methods (Supplementary Figure S10B). We were able to specifically amplify this i-tRF and to quantify its expression in 21 of the 22 experiments (Figure 10A). In five of the 11 analyzed pairs there was a statistically significant decrease in the tumor sample (*p*-val < 0.01; Student's *t*-test) whereas in two other samples, the fragment's expression was statistically significantly increased in the tumor (*p*-val < 0.01; Student's *t*-test). These results validate the existence of the novel i-tRFs in independent samples and provide initial evidence that such fragments have differential abundance in health and breast tumors, in agreement to what we deduced from analyzing the BRCA datasets. The second i-tRF, from the GlyTCC tRNA, spanning the anticodon triplet was quantified in eight different normal and breast cancer cell lines using the Multiplex miRNA assay (Figure 10B). In all of the cases, the i-tRF was detected and found present in the cell lines significantly above the background threshold.

We stress that our intent with these experiments was not to determine a “signature” of expression for the internal fragments at hand but rather to show that these i-tRFs are present in a variety of settings with relevance to breast cancer studies. Consequently, we analyzed datasets that correspond to different hormone profiles and come from patients representing two different races and for whom we have no knowledge of the specific population membership (such attributes have not been part of the typical questionnaire to date). With that in mind, and taking into account the dependence of fragment expression on population and race that we demonstrated in the earlier sections, the apparent diversity of expression shown in Figure 10 is expected.

## DISCUSSION

In this report, we described our analyses of the personalized transcriptomes of many hundreds of individuals and our discovery of links between molecules, the tRNA fragments, and attributes such as tissue, disease, disease subtype, gender, population and race. These links are important because the molecules, which are part of the personal transcriptome signatures of each individual, were shown to be functional, constitutive, and identically present across *like* individuals. However, these molecules exhibit statistically significant differences across individuals who are dissimilar in one or more of these attributes. These findings build on our earlier reports of race-dependent differences in pseudo-gene levels [46], and, of gender-/population-/race-dependent difference in miRNA isoform levels [47]. They also provide a new perspective for our recent findings that the nuclear genomes of humans, primates, and marsupials, but not rodents, are riddled with lookalike copies of mitochondrial tRNAs [35, 36]. Another important element of our study is the discovery of i-tRFs, an unsuspected and previously uncharacterized family of functional molecules, whose composition and abundance, as we showed, contributes much of the difference we observe across individuals. The findings suggest that these molecular differences that depend, among other things, on gender, population and race, will need to be taken into account when deciding a therapeutic regime.

With regard to tRNAs, our study contributes to the field's current knowledge about tRNA-derived fragments. We extended the concept of tRFs to include fragments from mitochondrial tRNAs, described novel length families for 5′-tRFs and 3′-tRFs, and discovered and characterized a novel class of *internal* fragments, the i-tRFs, that are wholly contained to the interior of the mature tRNA sequence and straddle the anticodon.

The project indeed required an interdisciplinary approach. The analysis of RNA-seq datasets at tRNA genomic loci imposed several constraints that needed to be taken into account and addressed. As we showed, exact multi-mapping on the whole reference genome is necessary as the sequences of tRNA genes have subtle differences and allowing mismatches during mapping will result in an ambiguous or erroneous deduction of the possible genomic origin(s) of each fragment. Using exact multi-mapping but mapping the sequenced reads on the tRNA space alone (and not on the entire genome) can also lead to erroneous results as there are multiple instances of partial tRNAs and tRNA-lookalikes in the human genome: if such non-tRNA loci are not taken into account, sequenced reads that may originate there would be erroneously treated as derived from *bona fide* tRNAs.

What is our definition of tRNA space? In this analysis, the tRNA space comprises all 610 tRNA genes of the nuclear genome contained in the tRNA-scan-SE database [54], all 22 tRNA genes of the mitochondrial genome, and the eight tRNA-lookalike sequences in the nuclear genome that are identical copies of 7 mitochondrial tRNA genes [35]. We are not aware of any previous studies that examined mitochondrial tRNAs for their potential to generate tRNA fragments. Thus, we included mitochondrial tRNA genes in our analyses. Doing so greatly complicates the ensuing analysis as the 22 mitochondrial tRNAs have hundreds of non-identical lookalikes across the human genome [35]. Their presence requires additional bookkeeping that would exclude any reads that map simultaneously on *mitochondrial* tRNAs and their *nuclear* non-identical lookalikes.

Inclusion of mitochondrial tRNAs in the analysis proved particularly fruitful, especially in light of our recently reported findings [35, 36]. In fact, it allowed us to demonstrate that the concept of tRFs extends beyond nuclear tRNAs, and to identify a significant number of statistically significant such fragments. Moreover, we showed that several of the mitochondrial tRFs exhibited race-dependent abundance profiles (Figure 6). The finding that mitochondrial tRNAs can serve as a source of fragments is important when considered in the context of mitochondrial biology. Mitochondria are involved in multiple cellular functions and are the point of convergence of many biological processes [55, 56], e.g. by conducting and regulating metabolic pathways, apoptosis [57] and signaling pathways [58, 59]. Importantly for our study, mitochondria have been linked to many diseases that are caused by mitochondrial tRNA dysfunction [60] and, presumably, a concomitant dysregulation of the corresponding tRFs. In this regard, the links between the abundance of full-length tRNAs and the abundance of fragments have not been characterized and await elucidation. Such links will be especially interesting in the context of diseases. For example, in breast cancer, and in cancer in general, tRNA levels have been shown to increase [61, 62]. On the other hand, as shown in Figure 8, the abundance of tRFs in tumor datasets exhibits a global decrease compared to the levels in normal tissue. This observation becomes particularly intriguing in the context of cancer biology considering that the fragments from the same anticodon have poorly correlated abundances (Figure 3).

The addition of the eight identical nuclear copies mitochondrial tRNAs to the reference tRNA space allowed for further expansion of our results. In both of the analyzed datasets, LCL and BRCA, the mitochondrial GluTTC anticodon produced the largest number of fragments. The respective tRNA gene has an identical (100%) nuclear copy on chromosome 5: had we not included this nuclear copy to the reference tRNA space we would have excluded a considerable number of tRFs. Naturally, many important biological questions arise that pertain to the possible biogenesis of these fragments; we examine these topics below. On the other hand, one should be careful not to include non-identical tRNA-lookalikes in the tRNA space: doing so is likely to lead to erroneous results as these loci may be evolutionary descendants that have diverged from their original tRNA function.

Another key technical point that had to be considered in our tRNA analyses relates to the fact that the genomic sequence does not capture the entire sequence of a mature tRNA. The nontemplated CCA addition at the 3′ end and the excision of tRNA introns are tRNA-specific processes that were taken into account explicitly when mapping the sequencing data. Among our derived fragments are ones whose sequence is shared by the tail of the first exon and the head of the second exon, indicating that these fragments arise from the mature or semi-mature tRNA molecule: such reads too need to be treated specially since they cannot be mapped on the full genome (and mapping on tRNA space alone is not advisable either, as explained above). One such example is the fragment trna14_TyrGTA_6_+_26569086_26569176@32.50.19__1_0_12, an *internal* fragment that maps solely on the 12 genes of the nuclear TyrGTA anticodon and spans the exon-exon junction in all 12 cases. This 19-mer, which does not appear elsewhere in the genome, would have been discarded if special provisions were not made for handling tRNA introns.

We emphasize that in this analysis we focused solely on fragments that are within the span of a mature tRNA. Consequently, we did not consider in our analyses any fragments that could derive from the precursor molecule, e.g. fragments that arise from the 3′ end of the precursor tRNA and are cut during maturation of the tRNA in the nucleus [5, 7]. Many aspects on the biogenesis of tRFs remain unknown, especially for the novel fragments that we report in this study, namely the new length-categories and the novel family of *internal* tRNA fragments. It is conceivable that there are multiple possible such pathways and that these can be independent for different types of fragments. Even though the existence of fragments that incorporate the nontemplated CCA addition and of fragments spanning two consecutive exons suggest a mature tRNA origin, extensive experimental work will be required for at least some of the fragments that we report in order to decipher the mechanisms and the cellular, molecular and environmental conditions that govern and drive the biogenesis.

Among the many uncovered fragments is a considerable-in-size group of mitochondrial tRFs. We point out that at least some of these fragments may not have mitochondrial-specific localization and function. Previously, mature mitochondrial tRNAs were shown experimentally to be in the cytosol [63]. However, the existence of exact tRNA-lookalikes in the nuclear chromosomes and the evidence that they are transcribed [35], in conjunction with the transcriptomic signatures that we reported in this study, complicate matters, especially in the context of human disease. Indeed, based on the currently available evidence it is conceivable that at least some these fragments may originate and function outside of mitochondria and possibly independent of mitochondria.

If mature tRNAs indeed serve as the source of the reported tRFs, then the base modifications in the mature tRNA are probably carried by the fragments. One such example was recently shown for a mitochondrial 5′-tRF where a known base modification caused specific sequence variations in the read sequence [38]. However, in humans, the global tRNA modification landscape is largely unknown making it currently impossible to comprehensively predict specific sites of variation. It is known that non-complementary nucleotides are frequently incorporated in cDNA synthesis during reverse transcription at the corresponding sites of modified bases [64, 65]. Mapping with mismatches is a double edge sword: it seemingly provides a solution to this problem but as we have demonstrated doing so will lead to biased and erroneous interpretations. An additional complication can emerge from the biogenesis of the fragments. At least a subgroup of them arises as a result of Angiogenin cleavage [10, 12, 22], an enzyme that leaves a cyclic phosphate at the 3′ end of the cleaved site [66]. The presence of the cyclic phosphate can hinder the ligation of the sequencing adaptors making such sequences ‘invisible’ to deep-sequencing approaches without any prior treatment. Nonetheless, some of the Angiogenin-derived fragments can be deep-sequenced indicating that some of the fragments are not 3′-modified. Indeed, previous studies [21, 38], as well as the current study, demonstrate that a significant amount of knowledge on tRNA biology and tRFs can be extracted from deep-sequencing datasets. It is currently not known under which conditions these modifications will occur or which fraction of the resulting fragments carry them. However, these modifications affect only the abundance of the observed molecules [21] and do not give rise to any sequencing artifacts [21]. This represents a true and present limitation in attempts to combine the fields of tRNA biology and RNA-seq; comprehensive future studies will be needed to address these matters.

It is important to emphasize that many of the fragments that we have discovered and described are distinct from what has been generically referred to as ‘tRFs’ in the literature of the last several years [5, 7, 17]. In fact, in addition to having discovered 5′ and 3′ products similar to the previously reported 5′- and 3′-tRFs, we also found that the 5′ and 3′ regions of mature tRNAs constitutively give rise to distinct molecular species of different and concrete lengths and different and concrete starting points (Figure 1). Moreover, we found a novel and very rich category of nuclear and mitochondrial tRNA fragments that are wholly *internal* to the mature tRNA sequence: this category includes fragments that begin in the loop region of the D-loop, long fragments that extend from the D-loop to the T-loop and straddle the anticodon, fragments that start at the anticodon and terminate within the T-loop, etc. One such *internal* molecule was previously described in the archaebacterion *H. volcanii* for the anticodon ValGAC and which gets cleaved at specific positions in the anticodon and the T-loop [32]. Instead, the *internal* tRNA fragments in our study arise from many anticodons, both nuclear and mitochondrial, originate from multiple yet consistent positions, while their origin and length attributes persist across individuals and are cell-type-dependent. Glimpses of the existence of i-tRFs were present in the early studies of the field with cell lines [15, 18] but neither research effort nor review publications pursued them until now or described them as a rich and distinct family of tRFs in eukaryotic organisms. We experimentally validated the existence of two members of this novel class of i-tRFs. Indeed, in independently obtained pairs of breast tumor and adjacent normal tissue from 11 different subjects, we specifically quantified the expression of an i-tRF and showed that for many of the pairs there were statistical differences between the normal and tumor tissue (Figure 10). In addition, we profiled eight different normal and cancer cell lines for a second i-tRF and found it present in all of them. Lastly, as evidenced from Figure 2, we have found many examples of fragments that have lengths and position similar to tRNA-halves but which are *not* stress-related and are produced constitutively across many hundreds of people.

Our analysis also revealed that different cell types have different tRF abundance profiles and this is evidenced by the existence of LCL-specific and BRCA-specific fragments. Although the LCL datasets are more deeply sequenced (2x) than the BRCA datasets, only half of the tRFs we discover in the BRCA dataset are present in the LCL dataset. Even if we confine ourselves to the common set of fragments, the two cell types/tissues (lymphoblastoid B-cells and the subset of normal breast tissue datasets) have distinct fragment abundance profiles (Figure 5). It thus follows that the lack of overlap in the identities of tRFs present in these tissues is unrelated to sequencing depth and instead reflects differences in the processes that give rise to and make use of these fragments in the two tissues.

Even when we restrict ourselves to the same tissue, we find that the abundance of tRFs is race-dependent; additionally, there are also glimpses of gender-dependent differences (Figures 6 and 7). The findings are in concordance with our previously reported race- and gender-dependent differences in the abundance of non-coding RNAs in the LCL datasets [47] and in platelets [46].

Looking at different tissue states we find the tRFs to have specific and distinct abundance profiles as well. This is exemplified by the results summarized in Figure 8: triple negative and triple positive tumors were found to have differences in tRNA fragment abundance both when compared to one another and when compared to the normal tissue. These cancers exhibit distinct clinical profiles, differences in survival rates and the patients have different treatment options [48, 67, 68]. In addition, it is becoming evident that triple negative BRCA exhibits race-specific clinical and molecular characteristics [69, 70]. The link between the tRF profile and clinical attributes as well as the mechanisms behind the differences in the profiles among cancer subtypes remain elusive, but our findings provide evidence of a possible involvement of the tRNA fragments in breast cancer biology.

The above findings clearly suggest a functional involvement of the tRFs in arguably important molecular events. The repertoire of the fragments' functional roles is probably not limited to a few cellular and molecular pathways, as the few tRFs that have been studied to date have been shown to regulate a diverse group of biological processes [5, 7, 17]. The possible functions may extend to novel and seemingly unrelated aspects of cellular mechanisms, as tRNA molecules have been shown to exhibit functional properties beyond translation [6, 71]. Focusing on the well-studied pathway of post-transcriptional regulation, we examined the Ago-loading characteristics of the fragments in the context of breast cancer. By using a dataset with multiple replicates of Ago CLIP-seq in different breast cancer cell lines [50], we were able to confirm Ago-loading for the classical 5′- and 3′-tRFs as well as for the newly-discovered i-tRFs. Also intriguing was our finding that the specifics of Ago-loading of these fragments depends on the cell line, and by extension on disease subtype. These data extend previous reports of association of the fragments with transcript regulation [14, 21, 49] while also paving the way for new functional analyses. As the shorter among the tRFs are sized approximately like miRNAs, it is reasonable to assume that such Ago-loaded fragments will be targeting other RNA transcripts; one such example was reported and studied recently [20]. Given the large numbers of fragments computational analyses in combination with the mining of Ago CLIP-seq data will prove useful in this context and are bound to uncover unknown aspects of post-transcriptional regulation. In this regard, we recently published a method for mining CLIP-seq data and deriving high-confidence miRNA-MRE heteroduplexes [72]. Our method is directly applicable to studying the potential roles of fragments with short tRNA fragments replacing miRNAs.

In conclusion, we have presented a comprehensive and detailed analysis of two large publically available datasets that revealed a rich repertoire of tRNA-derived fragments many of which have not been previously reported. In addition to uncovering a novel family of tRNA fragments that are wholly internal to the mature tRNA, we provided evidence for tissue-, cell-type-, cell-state-, population- and race-dependent abundance profiles for all three categories of tRFs, namely 5′-tRFs, i-tRFs and 3′-tRFs. Our results shine a new light on tRNA molecules and tRNA-derived fragments and provide previously unsuspected evidence that this ncRNA layer is highly-dynamic and highly-regulated, and by extension important. The current study may seem to contribute to an already chaotic genomic complexity. Our view is decidedly optimistic and parallels that expressed in [73]: a more comprehensive characterization of the molecular players in a given context represents an important contribution to our attempts to design better and more targeted drugs.

## CONCLUSIONS

By analyzing two large publicly available datasets, we establish that tRNA loci are a rich source of constitutive fragments with unexpected dependencies. In particular, we demonstrate that the profile of these tRNA fragments depends qualitatively and quantitatively on the race and the gender of the individual. We also demonstrate that the profile depends on cell type: cells of different types exhibit distinct tRNA fragment signatures. Specifically for the breast cancer samples we also demonstrate that this signature can discriminate between races and among histological subtypes. However, the tRNA fragment profile in a given tissue type and tissue state is identical across individuals that belong to the same gender, population and race, strongly arguing for the fragments' constitutive nature. A large contributor to these observed differences is a previously unreported and very rich category of constitutive tRNA fragments that are wholly internal to the sequence of the mature tRNA: using a recently published method we demonstrate for two internal tRNA fragments that they are present breast cancer and adjacent normal samples, and in breast cancer cell lines.

## MATERIALS AND METHODS

### Notation

To facilitate the discussion about fragments, we will be augmenting the notation that is used by tRNAscan-SE [54]. In particular, we tag the existing labels with fragment-specific information, namely the relative positions inside a reference tRNA and the number of appearances in other tRNAs of the same or different anticodons. For example, the augmented label trna116_GluCTC_1_-_145399233_145399304@23.45.23__1_0_8 refers to the tRNA fragment that has length 23 and spans positions 23 through 45 inclusive of the mature trna116 of GluCTC, the latter being located on the reverse (negative) strand of chromosome 1 between positions 145399233 and 145399304 inclusive. In those cases where more than one genomic tRNA loci can produce this fragment, we chose only one tRNA locus to serve as a source-proxy. The last three numbers of the augmented label that follow the double underscore capture the following information: a) the number of different anticodons that may give rise to this fragment (1 in the above example), the number of pseudo tRNAs that also contain this fragment sequence (0 in the example), and, the total number of genomic loci within the tRNA space (see below) that are possible sources of the fragment (8 in this case). Lastly, for fragments whose 3′ end is within the span of the terminal CCA we add the infix “CCA” before the double underscore as in, e.g., trna75_MetCAT_6_+_28912352_28912424@57.76.20.CCA__1_0_2.

### Defining the tRNA space

For the purposes of this study, we combined a) the 22 known human mitochondrial tRNA sequences (NCBI entry NC_012920.1 - http://www.ncbi.nlm.nih.gov/nuccore/251831106); b) 610 (508 true tRNAs and 102 pseudo-tRNAs) of the 625 nuclear tRNA sequences from gtRNAdb [54]; c) the eight genomic intervals chr1:+:566062-566129, chr1:+:568843-568912, chr1:-:564879-564950, chr1:-:566137-566205, chr14:+:32954252-32954320, chr1:-:566207-566279, chr1:-:567997-568065, and, chr5:-:93905172-93905240 that correspond to *exactly* identical instances (tRNA-lookalikes) of seven mitochondrial tRNAs TrpTCA, LysTTT, GlnTTG, AlaTGC (x2), AsnGTT, SerTGA, and, GluTTC respectively [35]. We excluded from the considered gtRNAdb entries the selenocysteine tRNAs, tRNAs with undetermined anticodon identity, and tRNAs mapping to contigs that are not part of the human chromosome assembly. In total, our reference tRNA space comprises 640 sequences.

### Mapping on the genome

The repeating nature of tRNA sequences requires that special steps be taken when mapping the RNA-seq data on the genome. In particular, we must:

- *allow for multiple hits*. Recall that any given tRNA anticodon may have instances at multiple genomic locations. To account for this and properly map sequenced reads arising from such loci we permit any given sequenced read to potentially map to up to 10,000 distinct genomic locations. See also below about excluding some of the mapped reads from further consideration.
- *seek only exact matches*. As is known, and independently of the platform that is employed, sequenced reads occasionally contain errors that are manifested in the form of nucleotide replacements, nucleotide insertions or deletions (indels), or various combinations thereof. Thus, to accommodate the possibility of such events a small number of indels and mismatches has typically been permitted during the mapping step of deep sequencing data: even though doing so may intuitively suggest more flexibility and improved mapping rates, when working with tRNAs this flexibility translates into localization errors. We thus use a conservative mapping strategy and allow only the exact mapping of reads on the genome without any insertions or deletions.
- *map on the full genome (and not on the tRNA space only)*. As we discussed in the Results section above, compiling a database of all known tRNA sequences and then mapping the sequenced reads to it would miss the fact that some segments of the known tRNAs also appear inside non-tRNA sequences, and lead to incorrect conclusions. We thus map the sequenced reads on the full genome then post-process each mapped read and discard those that map both inside and outside the known tRNAs.
- *take into account the presence of the terminal CCA*. Any sequenced reads that correspond to the 3′ of mature tRNAs will include the post-transcriptionally added terminal triplet CCA. Since we enforce strictly exact mapping of the reads, we cannot accommodate CCA's presence by allowing an adequate number of mismatches (replacements). Instead, and prior to mapping, we create a modified instance of the genome where we use CCA to replace the three genomic nucleotides immediately downstream of each of the 640 reference mature tRNAs.
- *account for introns considering that 31 of the bona fide tRNAs contain introns*. As our work focuses on mature tRNAs, we permit reads to span tRNA exon-exon junctions but discard reads that partially step on the intron segment of such tRNAs.

### Dataset normalization and statistical analyses

For each of the datasets, we built an expression matrix with each row representing a fragment independently of whether the fragment exists on multiple tRNA loci. Through a filtering step we excluded lowly expressed tRFs as well as fragments that were not supported by many datasets: for the LCL dataset collection, we required an expression of at least 30 reads in each of at least 30 of the datasets; for the smaller BRCA dataset collection, we required an expression of at least 30 reads in each of at least 20 datasets; and, for the Ago HITS-CLIP-seq dataset, we required an expression of at least 30 reads in each of at least 3 datasets. The raw expression value of each fragment was normalized using the sequencing depth of the respective dataset and the normalized value entered in the expression matrix. For the LCL dataset, the deep sequencing was shared by seven independent laboratories and, thus, we imposed a further normalization step: using the five samples that had been sequenced in all seven laboratories, we estimated an average expression vector for each laboratory, identified the laboratory with the most sequenced samples (laboratory 1), and rescaled each fragment's abundance by referring to this laboratory's data. Rank normalization was performed in each dataset independently. For each dataset, each abundance value was substituted with its position (rank) in the dataset. When two or more values were equal, they were replaced by the average of the ranked values that were to be attributed to them.

Statistical analyses were performed using the R statistical package [74]. PCA was run with the *prcomp* function and PLS-DA with the *plsDA* function of the *DiscriMiner* package [75]. For SAM, we used the *samr* package [44] with 5,000 permutations with the exception of the MDA-MB-231/BT-474 comparison where 720 permutations were performed. Heatmaps and hierarchical clustering of the correlation matrixes of the specific anticodons were done using the *heatmap.2* function of the *gplots* package. Hierarchical clustering was also performed with the *hcluster* function of *amap* package using the Kendall's tau coefficient as a distance metric and visualization was performed with the *dendextend* package.

For the comparison of the different breast cancer subtypes and normal datasets, we integrated the PLS-DA and SAM outputs. Specifically, for each of the comparisons with PLS-DA, we extracted the VIP (Variance Importance in Projection) scores and kept tRFs that had a VIP score of *at least* 1.5. The filtered fragments were also analyzed using SAM to identify differentially expressed fragments between categories at a strict FDR of 0.00% (Supplementary Figure S6 and Supplementary Table S4). As it is unknown whether the relative abundance of the lengths of the tRFs follows a particular distribution, we used the non-parametric Mann-Witney U-test to statistically evaluate the differences in the length histograms.

### Selective experimental amplification of tRFs

As our study uncovered a new category of *internal* tRNA fragments we chose the following fragment for further experimentation with dumbbell-PCR:

trna10_AspGTC_12_-_125424193_125424264@15.35.21__1_0_12

This fragment was sought in independently obtained breast tumor datasets whose hormone profile is provided in Supplementary Table S10. Total RNA was extracted from tissue samples using Trizol (Life Technologies). For the quantification of this fragment, we used a special method that specifically identifies RNA molecules with specific endpoints and can discriminate between tRNA fragments whose endpoints differ by as little as 1 nt. The method is described in detail in [51]. Briefly the protocol had as follows: 0.1 μg of total RNA was used for ligation with 20 pmol of a 5′ stem-loop adaptor by the T4 RNA ligate 2 enzyme (New England BioLabs). Following 30 min incubation at 37°C, 20 pmol of a 3′ stem-loop adaptor were added, incubated for 30 min and left for overnight ligation at 4°C. Using 1 μL of the resulting mix, qPCR was performed with the One-step PrimeScript RT-PCR kit (Clonetech) using 2 pmol of each of the qPCR primers and the specific TaqMan probe. The following served as negative controls: (1) qPCR with 10ng of total (unligated) RNA as template, (2) qPCR with no template, (3) ligation and wPCR with no template, i.e. only adaptors and primers mix. In all of the negative controls neither amplification signal nor a band at the target length on the electrophoreses could be detected. Electrophoreses were run on 3% MetaPhor Agarose gels (LONZA). QPCRs were run in triplicate and results were normalized to the U6 RNA using the 2(−Δ Δ C(T)) method [76]. It is noted that the C_t_ values for the tRNA fragments in the breast samples of Figure 10 ranged between 25 and 28 cycles. For U6 quantification, reverse transcription was performed with the SuperScript III Reverse Transcriptase kit (Life Technologies) and qPCR with the SYBR Select Master Mix (Life Techonologies). All used adaptor, probe and primer sequences are listed in Supplementary Table S11.

The following fragment was chosen for quantification in eight different model cell lines:

trna10_GlYTCC_17_+_8124866_8124937@31.53.23__1_0_8

using the FirePlex method (Firefly BioWorks). The cells were cultured in their specific culture media: DMEM (MDA-MB-231, MDA-MB-468), DMEM/F12 (MCF-10A), EMEM (BT-20, MCF-7), L-15 (MDA-MB-453, MDA-MB-157) and RPMI1640 (BT-549). RNA was extracted from each cell line using Trizol (Life Technologies) and was sent for quantification of the specific fragment with the Multiplex miRNA Assay (Firefly BioWorks) [52, 53]. Raw values are reported as the average of at least 10 independent measurements after subtracting the background noise.

## SUPPLEMENTARY MATERIAL FIGURES AND TABLES




