# Supplementary material for: Dissecting tRNA-derived fragment complexities using personalized transcriptomes reveals novel fragment classes and unexpected dependencies
Source: Oncotarget. 2015 Jul 6;6(28):24797–822. doi: 10.18632/oncotarget.4695 (PMC4694795; doi:10.18632/oncotarget.4695)
Supplement: Supplementary file 1 [file oncotarget-06-24797-s001.pdf]

Dissecting tRNA-derived fragment complexities using personalized transcriptomes reveals novel fragment classes and unexpected dependencies

Supplementary Material

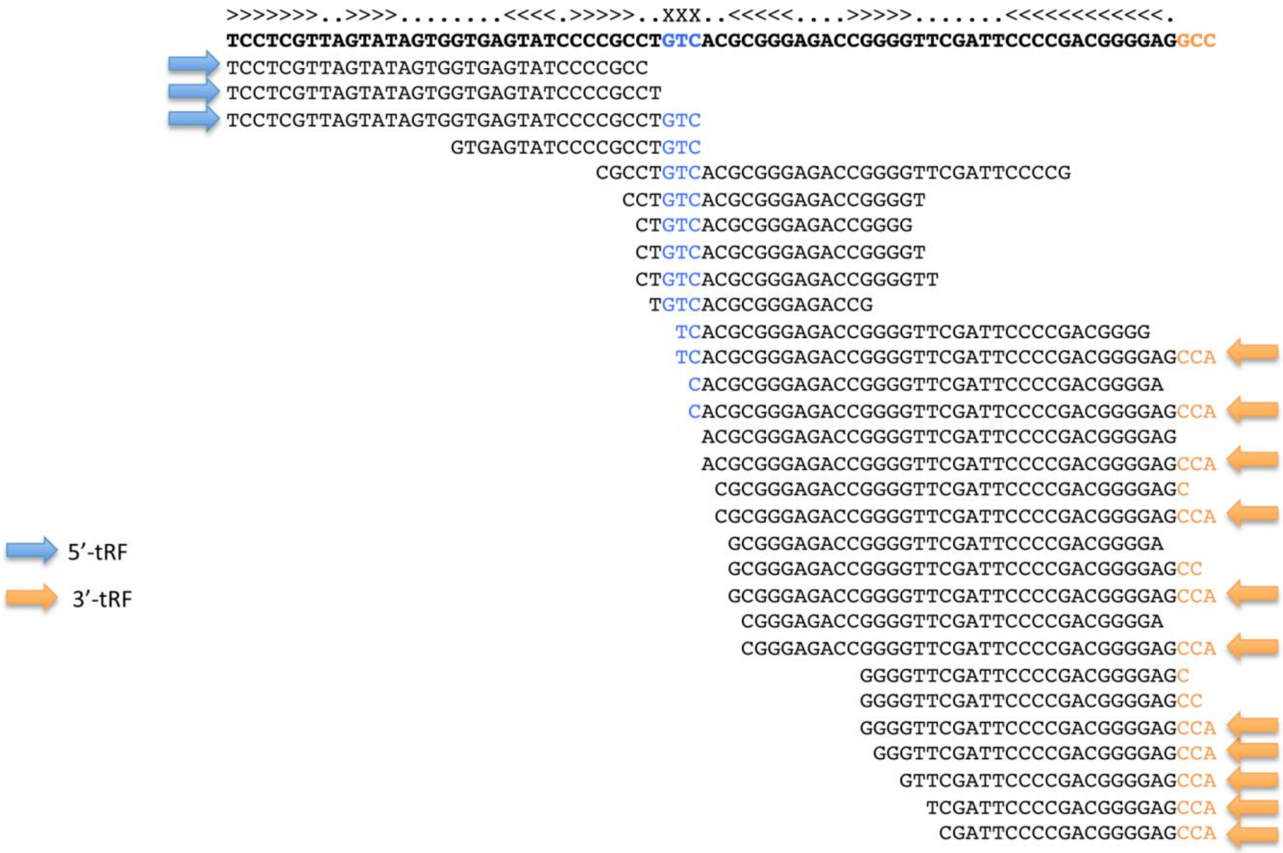

Figure S1: Schematic representation of the tRNA fragments mapped to the trna10 AspGTC locus on chromosome 12 (strand: “–”, coordinates: 125424193-125424264 inclusive) in the LCL dataset. The first sequence shows the genomic sequence of the tRNA gene. All lines below show the tRNA fragments found with significant expression in this study. Blue and orange arrows mark the 5´-tRFs and 3´-tRFs, respectively. The rest of the fragments are considered as i-tRFs. The anticodon is colored blue and the CCA tail in orange.

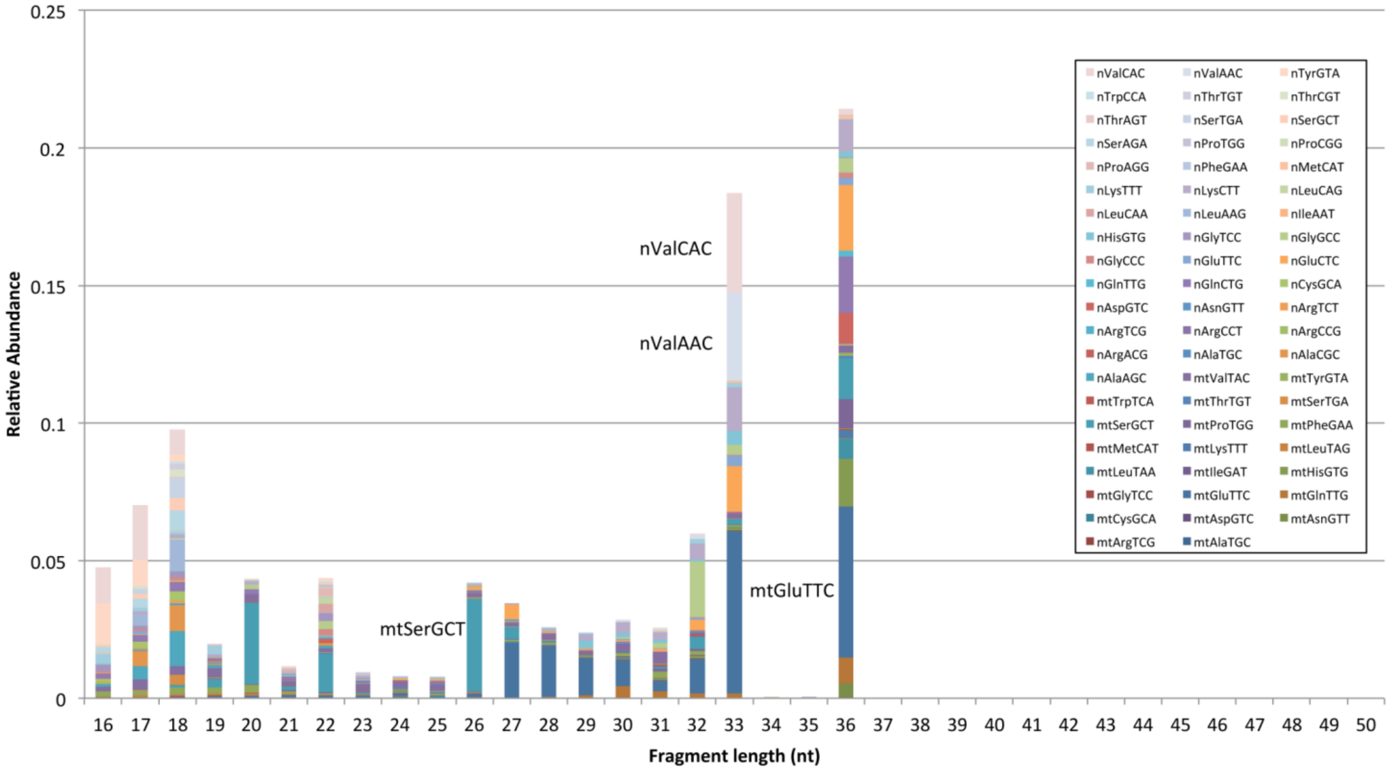

Figure S2: Abundance per fragment length per anticodon in the LCL dataset

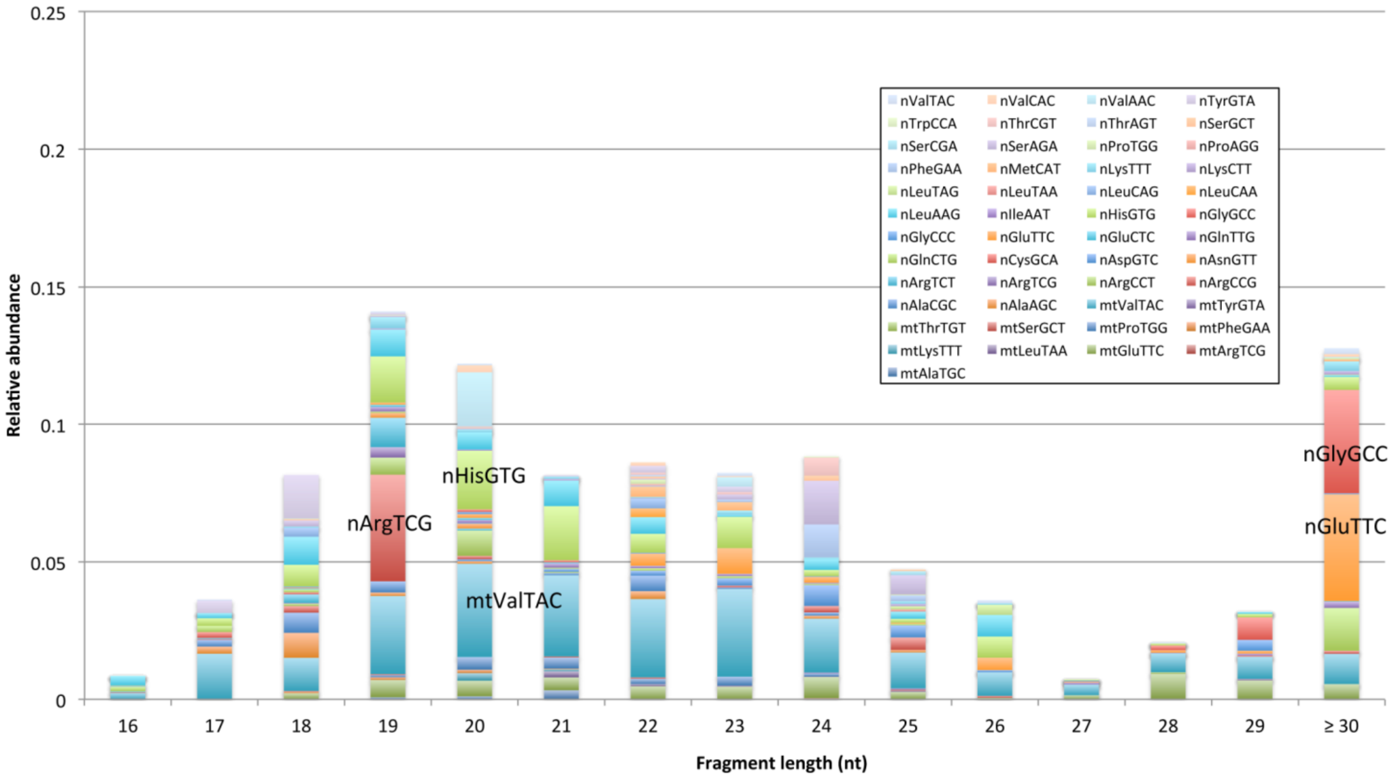

Figure S3: Abundance per fragment length per anticodon in the BRCA dataset

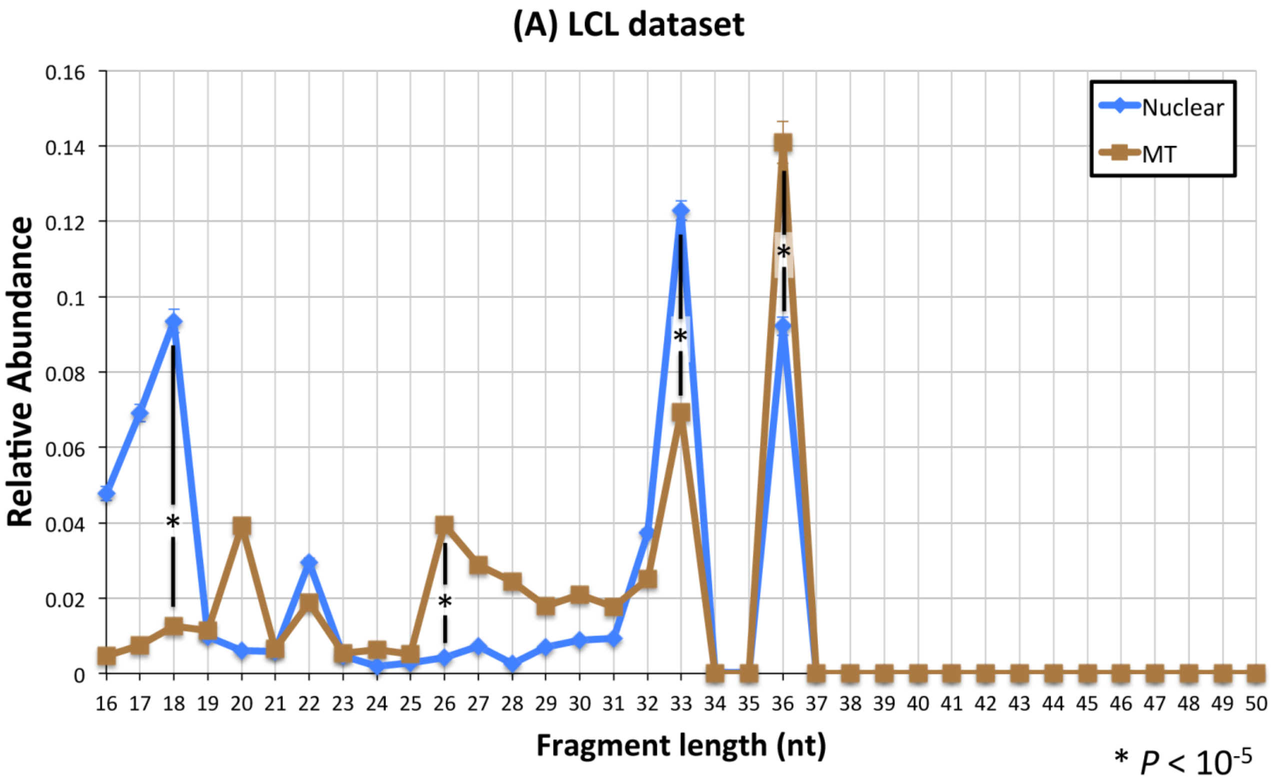

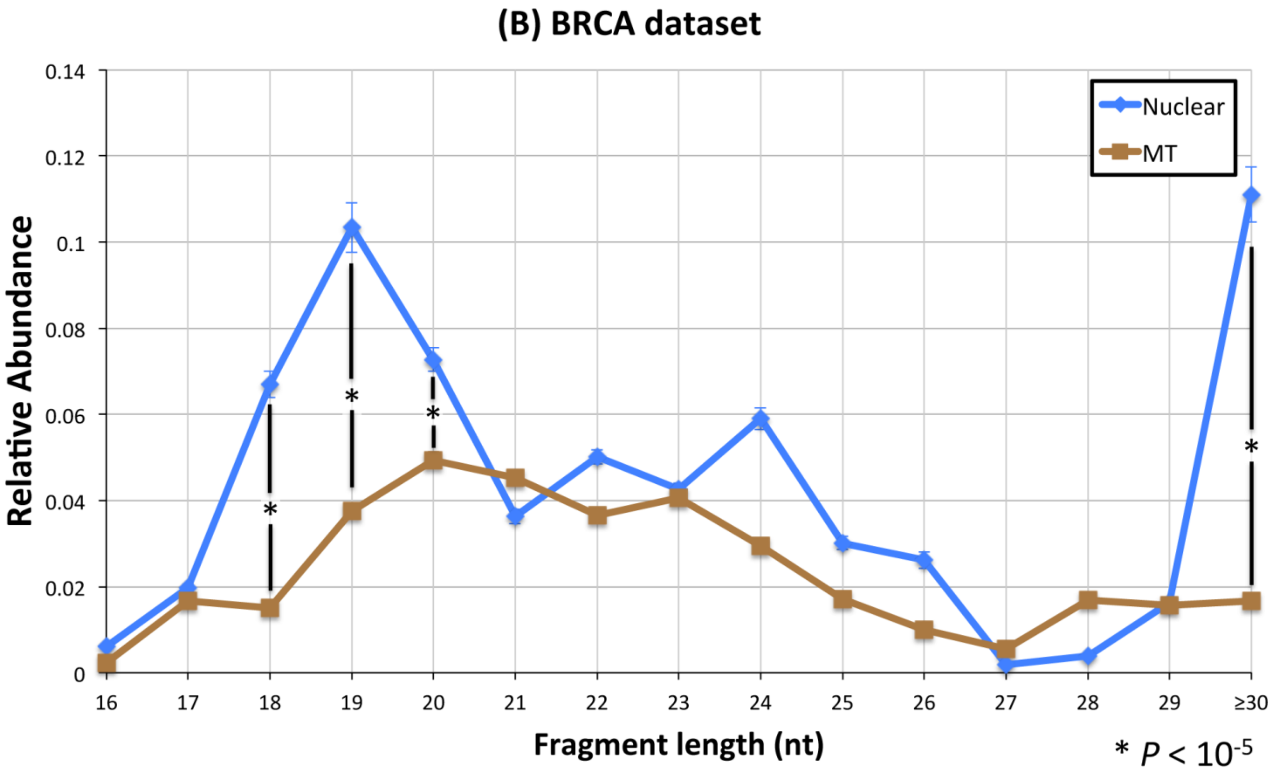

Figure S4: Relative abundance of fragments from nuclear and mitochondrial tRNAs as a function of their length for the LCL (A) or the BRCA (B) dataset. Error bars capture the standard error across 452 (A) and 311 samples (B). The statistically significant difference in abundance (P-value; Mann-Whitney U-test) is indicated for two cases in each dataset.

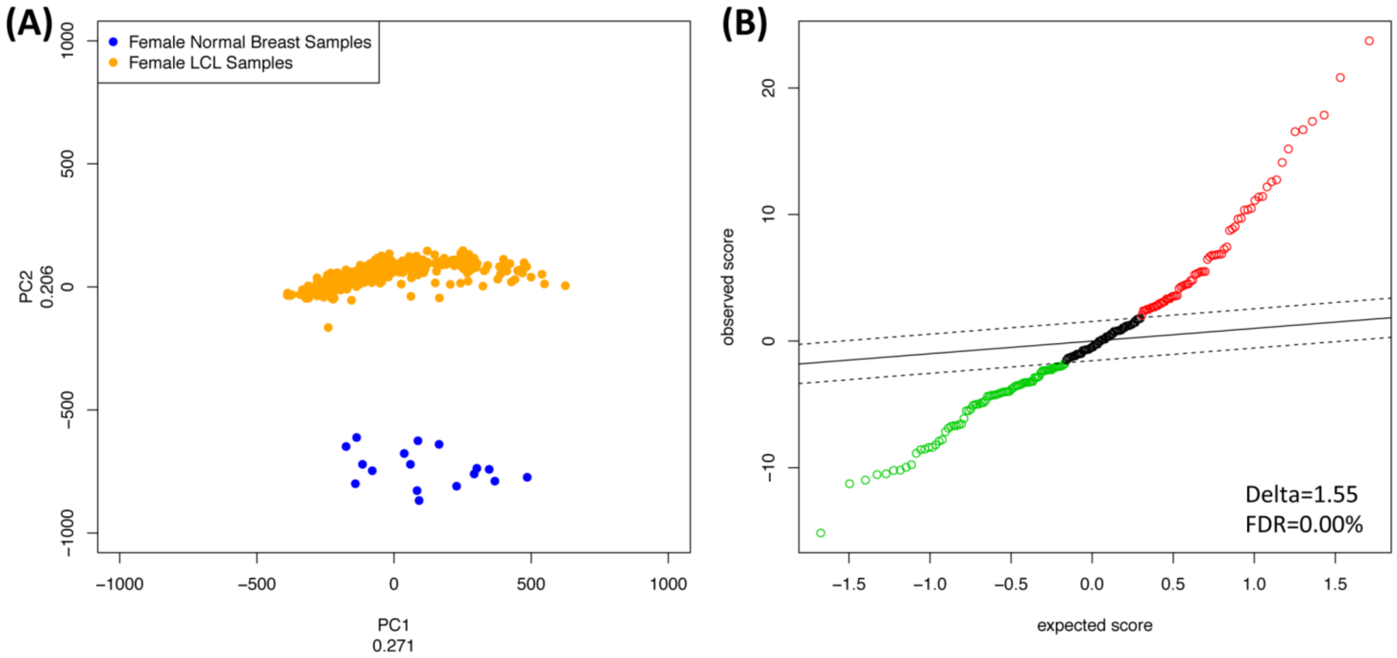

Figure S5: PCA analysis (A) and SAM (B) of the LCL and BRCA samples using only those tRNA fragments that are expressed in both collections. Data were ranked-normalized prior to analysis to exclude potential experimental quantification biases.

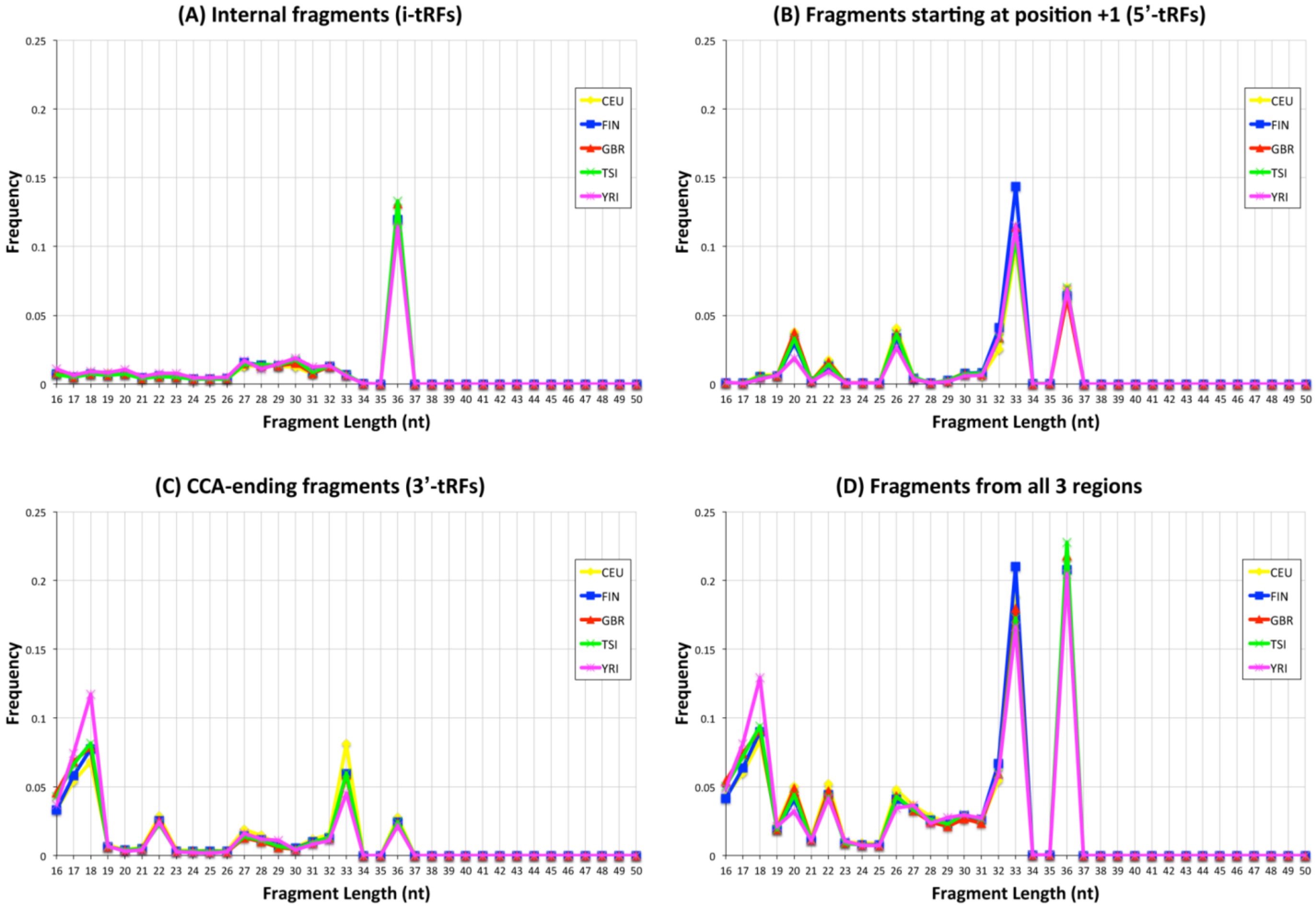

Figure S6: Distributions of fragment length per population in the LCL dataset

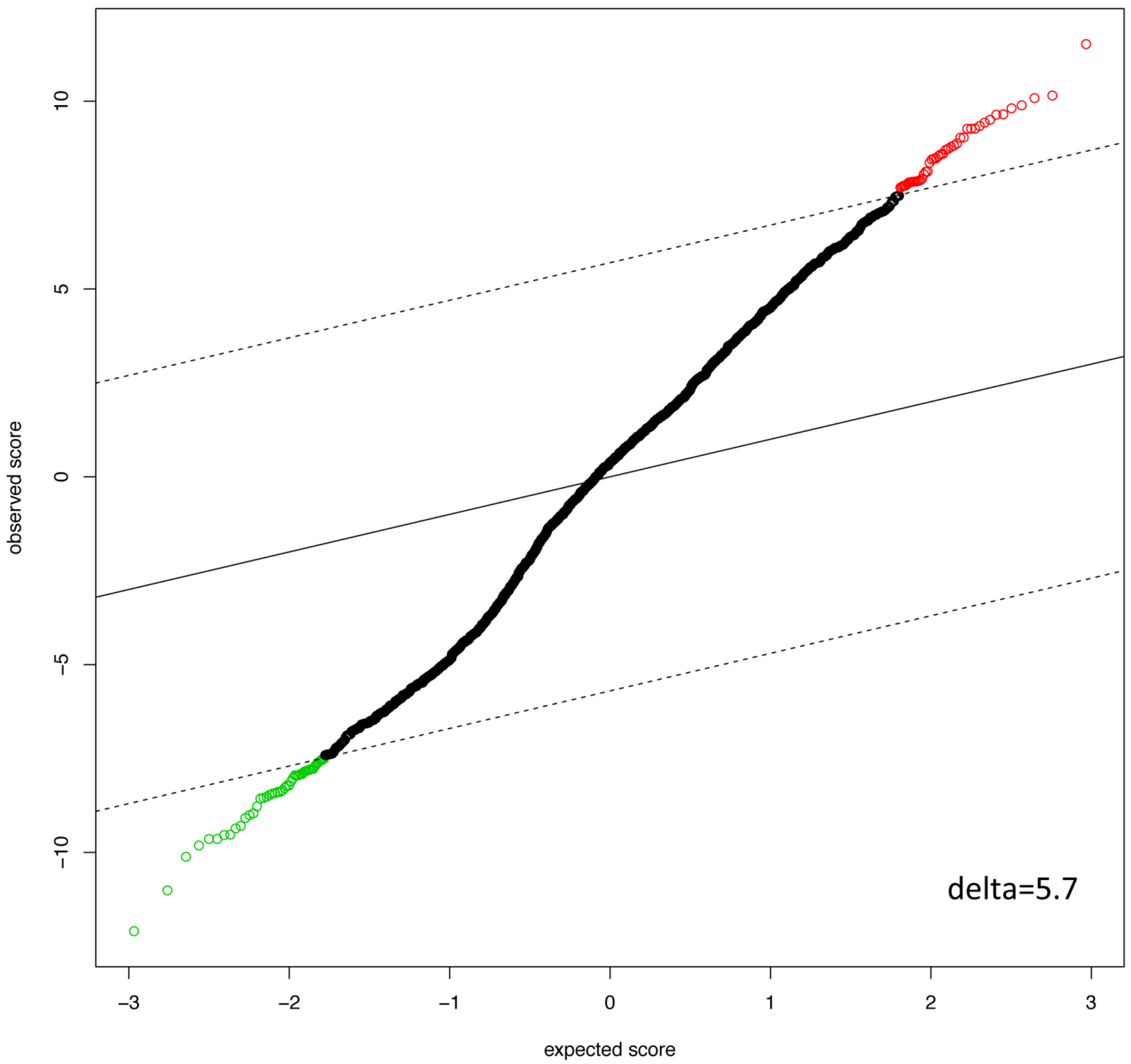

Figure S7: SAM graph for the YRI vs the CEU population comparison in the LCL dataset.

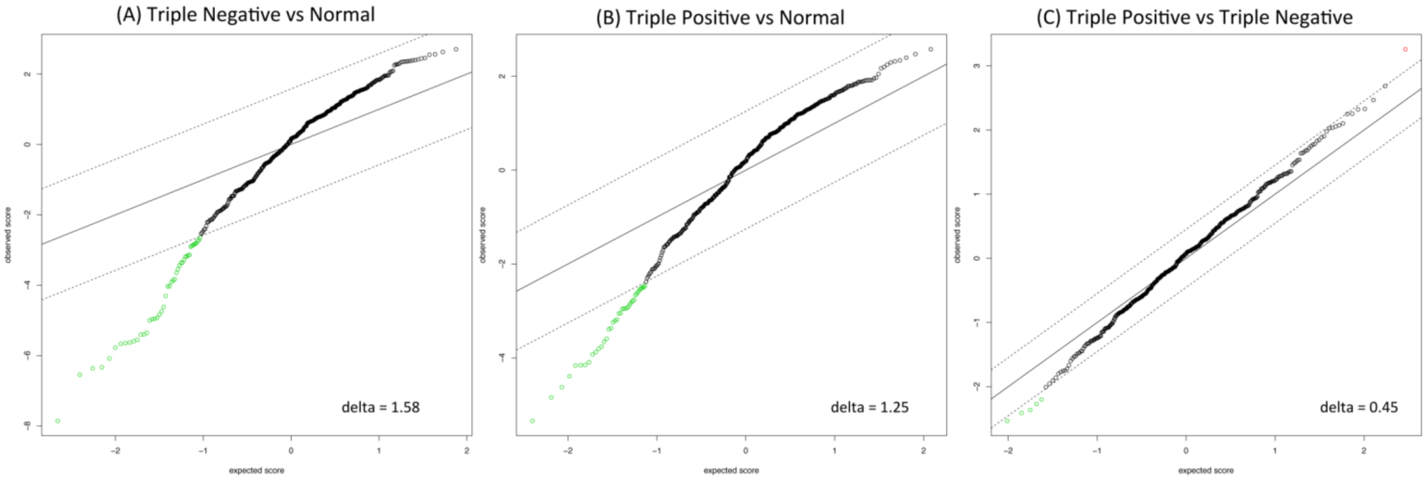

Figure S8: SAM graphs for the respective comparisons in the BRCA dataset.

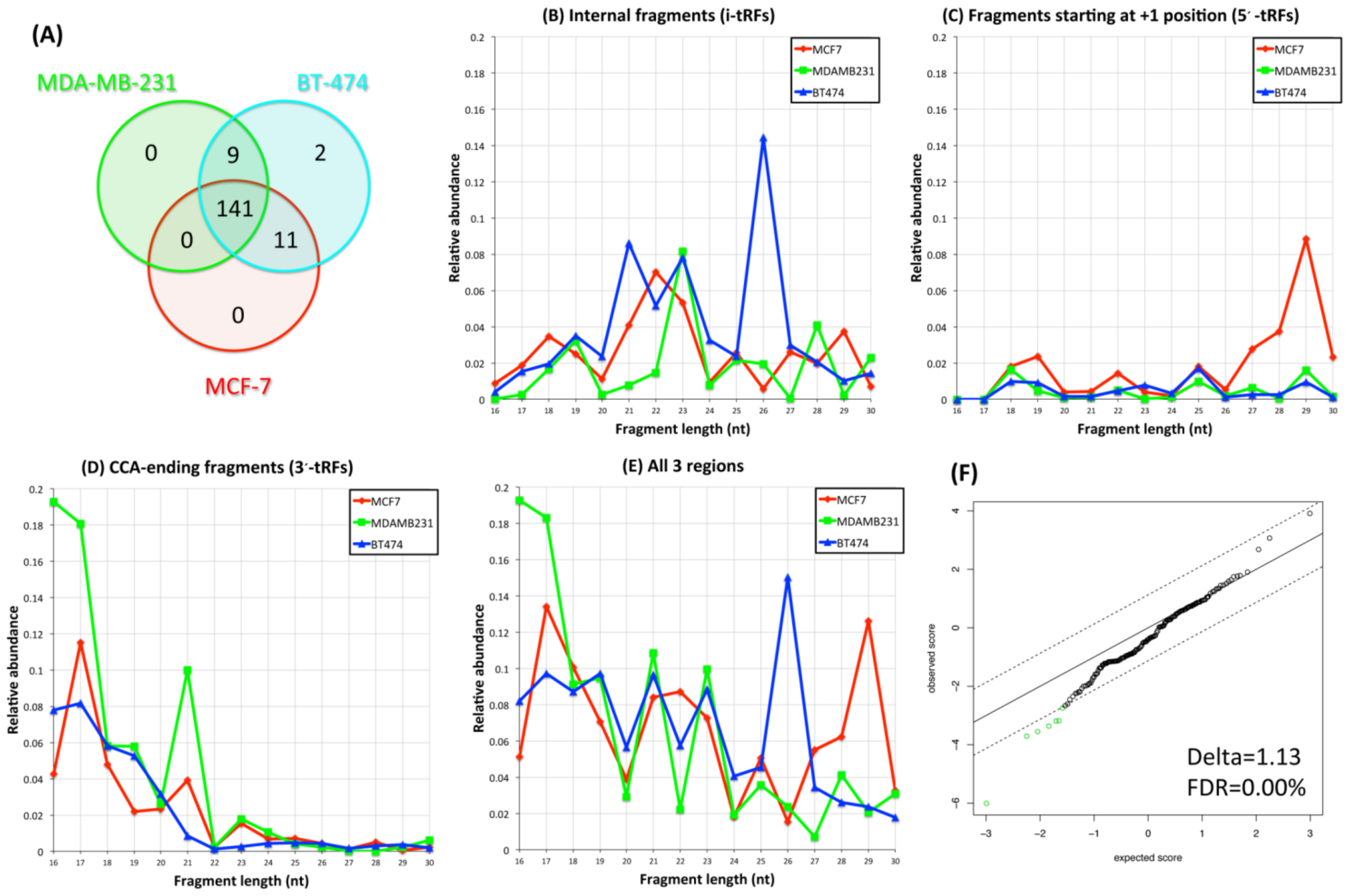

Figure S9: Differential Ago-loading of tRNA fragments in three breast cancer model cell lines. (A): Venn diagrams of tRFs in each cell line after filtering. Note that there were only two out of the 163 tRNA fragments expressed exclusively in one cell line indicating <u>quantitative</u> (and not qualitative) Ago-loading differences among the cell lines. (B-E): Length distributions of the internal tRNA fragments (B), of the 5´-tRFs (C), of the 3´-tRFs (D) and all the tRFs (E). (F): SAM plot for the comparison of the MDA-MB-231 vs the BT-474 Ago-loaded tRFs.

(A)

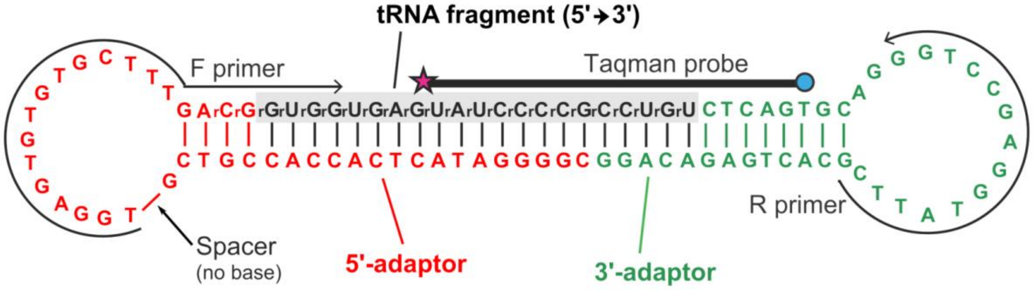

(B)

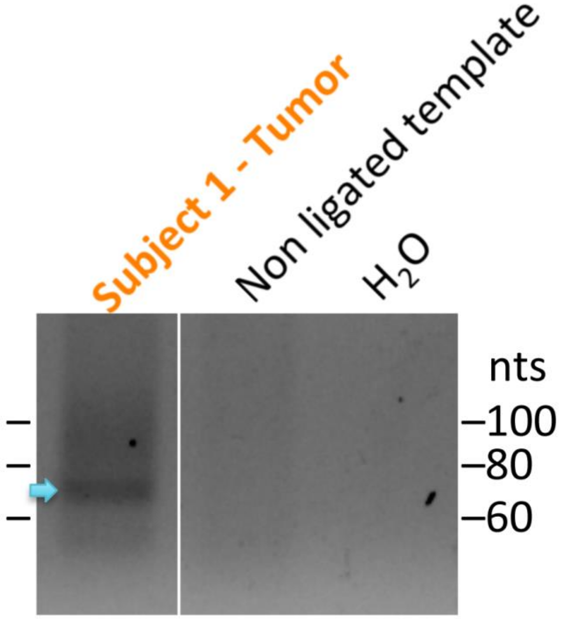

Figure S10: (A): Representation of the special PCR method to detect and quantify the tRNA fragment. The fragment and specific adaptors sequences are colored with different colors. The letter 'r' before each base indicates that the pentose sugar is a ribose (RNA). The "Spacer" indicates that no nitrogenous base is attached on the pentose backbone. (B): Agarose gel electrophoresis of the PCR product from the tumor sample of the patient "Subject 1" (first lane), of the PCR with substrate non-ligated RNA (second lane) and with no substrate (third lane). Only in the first case the tRNA fragment could be detected (cyan arrow).

<u>Supplementary Tables</u>

Table S1: Total number of possible tRNA fragments

Table S2: Raw data of the LCL dataset

Table S3: Raw data of the BRCA dataset

Table S4: Number of fragments observed in the LCL dataset

Table S5: Number of fragments observed in the BRCA dataset

Table S6: Distribution of fragments in true and pseudo tRNA genes

Table S7: Differentially expressed tRNA fragments in the LCL dataset as compared to the BRCA dataset.

Table S8: Differentially expressed tRNA fragments in the YRI compared to the CEU population

Table S9: tRNA fragments important for the PLS-DA model for the separation of the two genders in the YRI population

Table S10: Differentially expressed tRNA fragments between normal breast tissue and breast cancer subtypes

Table S11: Raw data of dataset from Pilai et al.

Table S12: Statistical significant differences in the MDA-MB-231 with the BT-474 Ago-loaded tRF profile as compared from Pilai et al.

Table S13: Characteristics of the breast cancer samples used for the independent experimental validations

Table S14: Primer, adaptor and probe sequences for experimental validation
